# A Six-Week Student-Led Project Designed to Provide Insight into Modern Photochemistry Research

**DOI:** 10.1021/acs.jchemed.4c01241

**Published:** 2025-03-06

**Authors:** Dominic Taylor, Leonardo Amicosante, Luize M. Luse, Martin R. S. McCoustra, Lee McMahon, Scott J. Dalgarno, Filipe Vilela

**Affiliations:** School of Engineering and Physical Sciences, Heriot-Watt University, Riccarton, Edinburgh EH14 4AS, U.K.

**Keywords:** Upper-Division Undergraduate, Organic Chemistry, Chemical Education Research, Hands-On Learning, Collaborative, Photochemistry, Synthesis, Computational

## Abstract

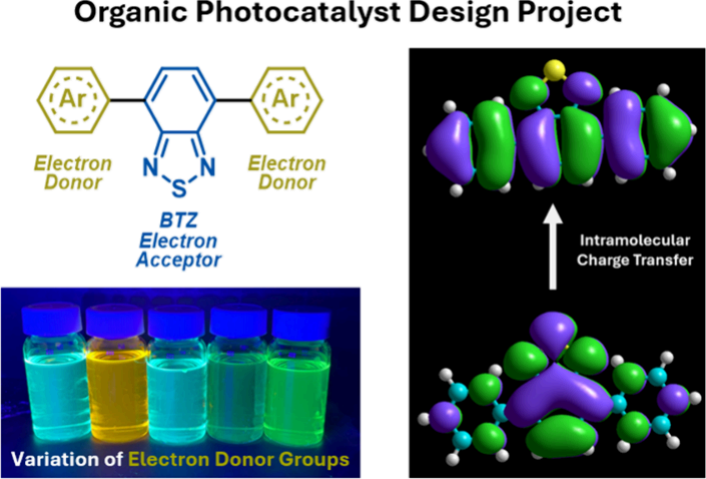

An advanced chemistry laboratory project has been developed to introduce final year undergraduate students to organic photocatalyst design using benzo[*c*][1,2,5]thiadiazole (BTZ) photocatalysts, prepared via a single Suzuki-Miyaura cross-coupling from cheap starting materials, as the example. This project took on a research lab style structure, and over the course of 6 weeks students were tasked to (1) synthesize and study the photophysical properties of three BTZ-based photocatalysts; (2) apply the BTZ photocatalysts to a test photoredox reaction using cheap home-built LEDs, and (3) perform rudimentary computational calculations to rationalize key experimental results.

## Introduction

**Figure 1:**
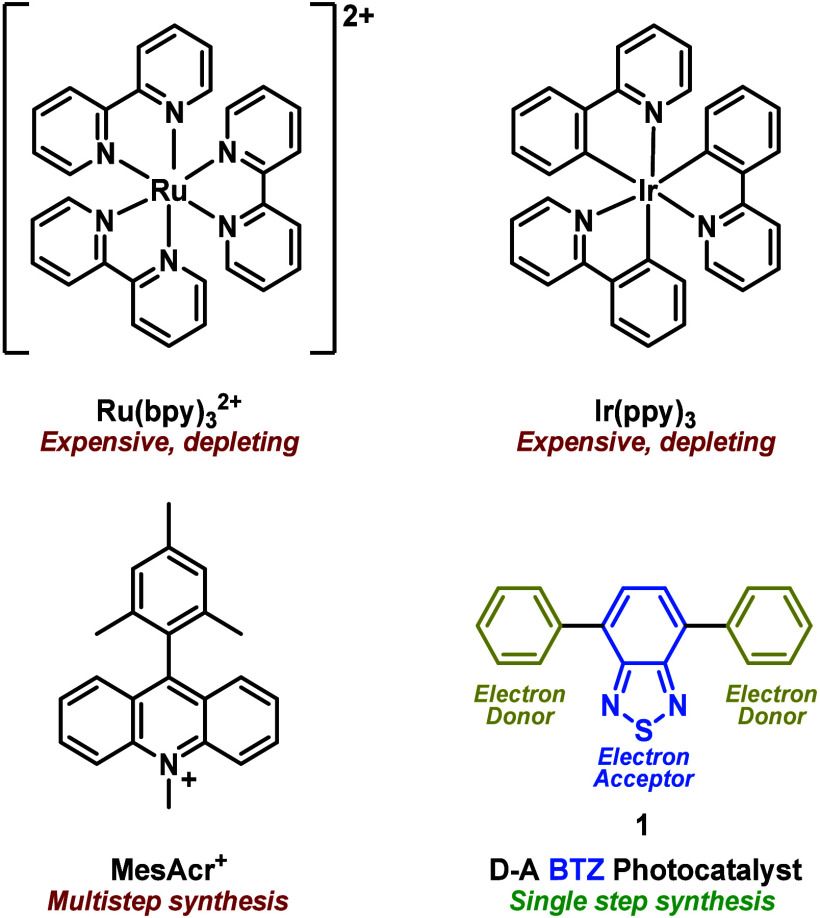
Chemical structures of selected photoredox catalysts prevalent in the literature. An example of a **BTZ** photocatalyst (**1**) is also shown. The electron donor phenyl groups and electron acceptor **BTZ** group of **1** have been highlighted.

In recent years, photocatalysis has emerged as a powerful tool for enabling chemical transformations.^1^ The success of a photocatalytic reaction is heavily underpinned by the properties of the photocatalyst, as it is responsible for engaging in single electron transfer with reagents. State-of-the-art photoredox catalysts are generally based on iridium or ruthenium polypyridyl complexes (such as Ir(ppy)_3_ and Ru(bpy)_3_^2+^), although their high cost and the natural scarcity of Ir/Ru raise questions over their long-term sustainability (Figure 1).^2^ Significant research has been conducted into organo-photocatalysts as alternatives, such as acridiniums salt (MesAcr^+^), although these can often require multistep syntheses.^3^

The prominence of photocatalysis in current chemistry research has led to the development of new undergraduate laboratory experiments including analyzing and testing photocatalysts,^4−7^ or exposing the students to new emerging technologies such as continuous flow photochemistry.^8−10^ However, there are very few reports of extended research projects that provide students with a holistic experience of the steps involved in the design of new classes of photocatalysts. The use of course-based undergraduate research experiences (CUREs) has been utilized across many areas of chemistry to provide students with a research-led environment.^11,12^ These projects diverge away from traditional “cookbook” teaching laboratory experiments and instead utilize a research laboratory style environment that offers the students greater insight into research life.^13^ One such example has been published by Nicewicz and co-workers, who detailed a CURE based on pyrylium salts as photoredox catalysts.^14^ However, there is a general lack of reported educational projects that combine the synthesis and testing of photocatalysts with computational modeling of their structural and electronic properties.^15,16^

**Scheme 1:**
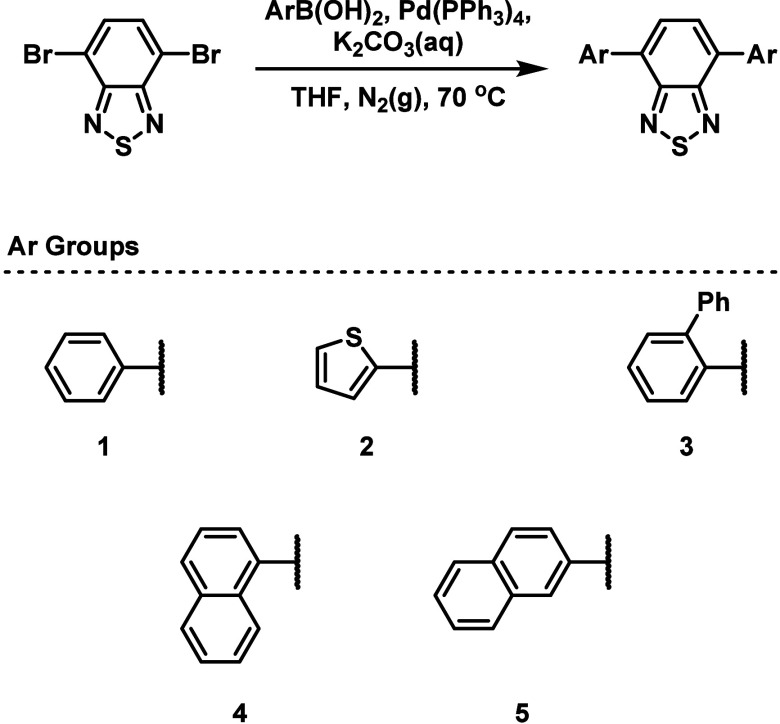
Synthesis of **BTZ** Photocatalysts **1**–**5** via Suzuki-Miyaura Cross-Coupling

Herein, we outline a six-week undergraduate research experience intended to give students a holistic view of the steps involved in the design, synthesis, and implementation of a new class of organo-photocatalysts. To provide the students with the most realistic experience possible, we elected to directly base the project on our recently published work concerning electron donor–acceptor **BTZ** photocatalysts (Scheme 1).^17^ These could be prepared via a single Suzuki-Miyaura cross-coupling from 4,7-dibromobenzo[*c*][1,2,5]thiadiazole (**Br**_**2**_**BTZ**) using commercially available aryl boronic acids: the wealth of inexpensive and commercially available boronic acids provided a convenient and cost-effective route through which the optoelectronic properties of the photocatalyst could be adjusted.

Part of the challenge in designing this undergraduate research project was translating cutting edge academic research into a condensed project that provided an accurate impression of research laboratories as part of an accessible learning experience for the students. Discussion on this subject will highlight issues such as accessibility/difficulty, the overall student learning experience where appropriate, and how these challenges were addressed. In particular, as photochemistry often makes use of expensive LED modules that can present a significant financial barrier to introducing photocatalysis into teaching laboratories, we constructed home-built LEDs that helped to significantly reduce the cost of running this project.

## Pedagogical Goals

On the surface, the aim of this research project was for final year undergraduate students to gain practical experience in the design, synthesis, and application of organic photocatalysts. More specifically, this project involved adaptation of contemporary research into a guided inquiry teaching experience that provided students insight into life as a research chemist. Within this broad objective, five key learning outcomes for the students can be identified:

1. To use a Schlenk line to carry out air-sensitive Suzuki-Miyaura cross coupling reactions.
2. To safely conduct photocatalytic experiments using LED modules.
3. To perform computational chemistry calculations on their photocatalysts to obtain information about their chemical and electronic structure.
4. To design experiments, perform risk assessments (with appropriate oversight), and collate experimental results.
5. To disseminate results by presenting their project findings through a written report and a social media poster.

## Results and Discussion

### Photocatalyst Synthesis and Characterization

This project was designed to be completed over a period of 6 weeks as part of a laboratory course for advanced chemistry undergraduate students (Year 4 BSc (Hons) students) and has been run for a total of three years at Heriot-Watt University. The students were divided into three groups of five, although this project could be run with smaller groups, if desired. The structure of this project purposefully diverges from the academic-led environment of fundamental teaching laboratories, instead placing the onus for project management more on groups of students as researchers.^12^ The students were provided with a laboratory guidebook that provided a brief introduction to the course and explained the structure of the project: this guidebook has been provided in the Supporting Information. A demonstrator was also on hand during each laboratory session to help the students if needed and to oversee health and safety.

To break the assignment down into more manageable goals, the project was split into three two-week blocks dedicated to (1) photocatalyst synthesis and characterization; (2) photoredox catalysis; and (3) computational studies, with a total of six 3 h laboratory sessions per two-week block. The specific work schedule within each two-week block was left up to each group to impart personal responsibility for their own research, allowing them to gain experience in designing their own experiments, conducting relevant risk assessments, and conducting those experiments in a safe, assisted environment.

The first 2 weeks of the project were dedicated to synthesizing the **BTZ** photocatalysts (Scheme 1): each group was tasked to synthesize a total of three photocatalysts (**1**, **2** and one unique option from **3**–**5**). This was achieved by carrying out a Suzuki-Miyaura cross-coupling reaction using **Br**_**2**_**BTZ** (1 mmol) and 2.5 equiv of an aryl boronic acid (2.5 mmol) (Scheme 1) using tetrakis(triphenylphosphine)palladium (0) as the catalyst. The guidebook for the course contained an example Suzuki-Miyaura cross-coupling procedure for the synthesis of a **BTZ** photocatalyst that was not a possible synthetic target for the students. The students were able to adapt this procedure to suit their own synthetic targets by changing the cross-coupling partners and the quantity of chemicals used. The example procedure also served to demonstrate to the students how to report the synthesis of chemicals and their characterization data in a suitable scientific manner.

Synthesizing the **BTZ** photocatalysts through Suzuki-Miyaura cross-coupling afforded an opportunity to teach the students basic Schlenk line chemistry techniques including how to evacuate and backfill reaction flasks with inert gases. The students were first instructed on how to carry out the Suzuki-Miyaura cross-coupling reaction by an in-person demonstration. This was supplemented through a video of a demonstrator performing the reaction that was made available to the students through the course intranet page: the students were directed to these videos through the laboratory guidebook and by the demonstrators. These videos were provided not only to help the students learn the new techniques but also in case a student was unable to physically attend class due to COVID-19 restrictions. These videos have also been made available on YouTube *in lieu* of being provided as Supporting Information files:

**Suzuki-Miyaura cross-coupling reaction:**https://www.youtube.com/watch?v=EyilEmKbux4

**Photochemical Minisci reaction:**https://www.youtube.com/watch?v=30Z7LQcK3yI

To determine if the Suzuki-Miyaura cross-coupling reaction was complete, the students were instructed to withdraw a sample of the reaction mixture under positive nitrogen flow, evaporate the solvent, and then perform a crude ^1^H NMR of the residue. For each photocatalyst, key peaks could be identified to provide an approximate measure of the completeness of the reaction (Figure S3). This is advisible, as partial reaction can result in a mixture of up to three products that cannot be separated easily. If the reaction was taken to completion, then purification could be achieved either through recrystallization or washing the crude solid with hot ethanol, depending upon the photocatalysts solubility.

Overall, the students were able to isolate their target photocatalysts with yields comparable to those of both the course instructor and an independent checker (Table 1). A typical time for full completion was 1–3 days depending upon the boronic acid used, with more electron rich examples reaching completion quicker. The use of a slight excess of boronic acid is favorable to force the Suzuki-Miyaura cross-coupling reaction to completion—this excess can be removed during the purification steps. Due to time constraints, the scope of potential electron donor groups available to the students was intentionally narrowed in favor of photocatalysts that offered interesting structure–property relationships. Although the photocatalysts used in this mini-project so far were all vetted, the range of commercially available boronic acids leaves room for students in future iterations to be potentially tasked with targeting novel structures as part of a CURE.^12^

**Table 1:** Comparison between the Instructor, Checker, and Student Yields for the Synthesis of Photocatalysts **1**–**5**

Compound	Instructor Yield (%)^a^	Checker Yield (%)^b^	Average Student Yield (%)
**1**	60	58	61
**2**	67	67	58
**3**	64	70	88
**4**	87	74	74
**5**	86	79	71

a Instructor refers to a 3rd year PhD student demonstrator.

b Checker refers to an undergraduate student tasked with independently verifying results.

**Figure 2:**
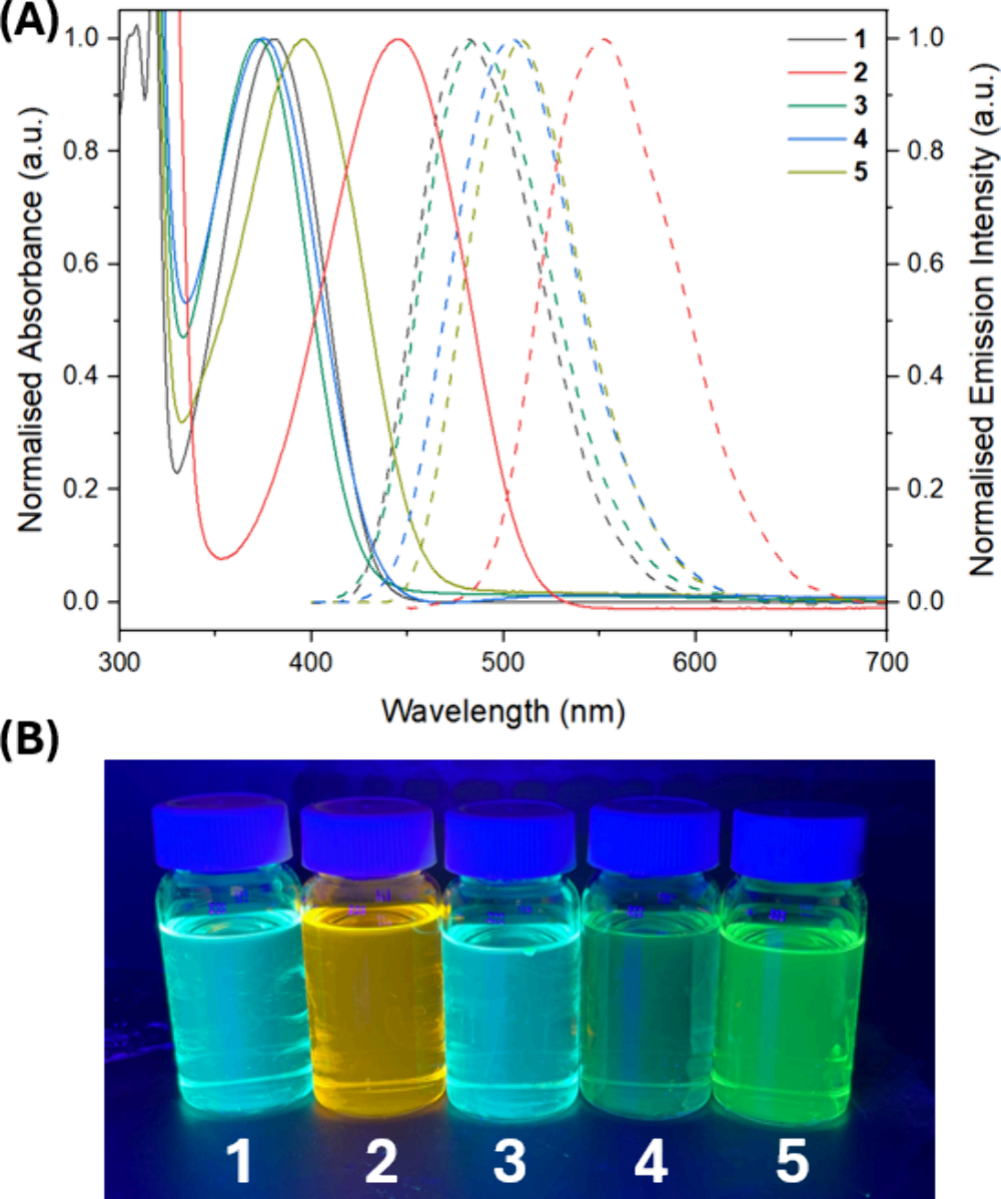
(A) Normalized UV–visible absorption (solid line) and emission spectra (dashed line) of **1**–**5** recorded in a CHCl_3_ solution. (B) Solutions of **1**–**5** (left to right) of arbitrary concentration in CHCl_3_ solution under UV-illumination.

Following synthesis, the students fully characterized their photocatalysts, which included recording ^1^H and ^13^C NMR spectra, IR spectra, as well as UV–visible absorption and emission spectra (Table 2). The expected absorption and emission spectra for photocatalysts **1**–**5** are provided in Figure 2A. One feature to note is that the students can easily visualize the changes in the photocatalyst emission by shining a UV light source (such as a TLC visualizer) onto a solution of their photocatalyst, which will result in intense fluorescence (Figure 2B). At this point, the students can begin to rationalize the effect of the photocatalyst structure on its absorption/emission properties such as the electronic effects of different electron donor groups on the HOMO–LUMO separation, or the disruption of π-conjugation by sterically bulky group (although this was easier to see via computational studies).

**Table 2:** Photophysical Properties of **1**–**5**

Compound	λ_abs,max_/nm^a^	λ_em,max_/nm^a^^b^	Stokes Shift/nm
**1**	380	482	102
**2**	446	552	106
**3**	373	486	113
**4**	376	504	128
**5**	396	509	113

a Recorded in CHCl_3_ solution.

b Excitation wavelength was set equal to the wavelength of maximum absorption for each photocatalyst.

### Photocatalyst Testing

**Scheme 2:**
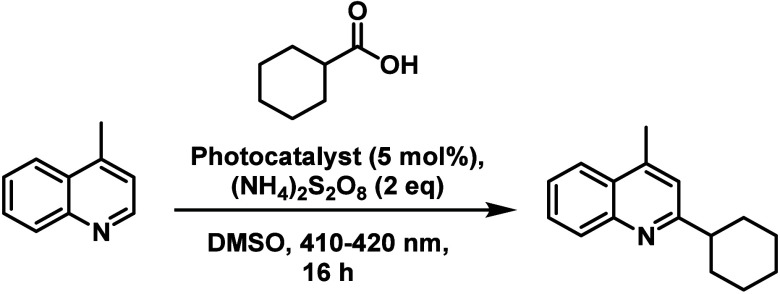
Minisci-Type Photocatalytic Coupling of Lepidine and Cyclohexane Carboxylic Acid

The photoredox reaction that was selected as a model test reaction was the decarboxylative C–H functionalization of lepidine using cyclohexane carboxylic acid and ammonium persulfate as the sacrificial oxidant (Scheme 2).^18^ This reaction was selected on the basis that the reaction conversion can be determined via ^1^H NMR spectroscopy following workup, and that all reagents are commercially available and cheap (Figures S4 and S5). The LED modules that were used for this project were constructed in house using six 3 W LEDs (410–420 nm) purchased from Future Eden Ltd. mounted onto an aluminum heat sink using thermal paste (Figure S6). This helped to drastically reduce the cost of the project by avoiding the purchase of the expensive premade LED systems that are typically utilized in academic research (see cost analysis below).

As was the case with the Suzuki-Miyaura cross-coupling reactions, demonstration of the photochemistry techniques and a supplementary video was provided to the students. To foster critical thinking, the students were afforded freedom with respect to how they tackled optimizing the photocatalytic reaction. The laboratory guidebook prompted the students to investigate the effect of reaction variables (such as changing the solvent, sacrificial oxidant, time, light distance, *etc*.) but did not indicate in what way they should be changed or which variables would be the most significant to the reaction conversion. The students were also prompted to perform control experiments: the key here was for the student to realize the role that each component played in the reaction. The photoactivity of the series of **BTZ** photocatalysts prepared by each group could then be tested under their optimized reaction conditions. An example of a set of conversions obtained by the students was 64, 2, and 70% after 20 h when utilizing photocatalysts **1**, **2** and **3** at 5 mol % loading, which was consistent with previously reported performance of these species.^17^ From these results, the students can observe differences in the activity of each photocatalyst and begin to form hypotheses that could be later supported by computational calculations. One factor in the rate of **1**, **2** and **3** is the presence of steric bulk on the electron donor group, which could hinder internal rotation as a nonradiative relaxation pathway that could otherwise deactivate photoexcited states.^19^

### Computational Studies

Rudimentary computational calculations were performed using Chem3D Pro and Hyperchem 8.0: these were programs that the students had prior experience using as part of earlier undergraduate teaching laboratories. As such, instructions on how to use the program were not required, although a demonstrator was available if the students needed assistance setting up their calculations. Chem3D Pro was used to generate MM2 optimized molecular geometries and measure the energy barrier for rotation of the aryl electron donor groups relative to the **BTZ** group (Figures S7–S11). A key observation that the students can draw from these results is that different electron donor groups present different structural properties for **1**–**5** that can impact the degree of π-conjugation present. The optimized energy minimized structure of compound **1** is a nonplanar geometry with an angle of approximately 30° between the **BTZ** and phenyl groups. Performing a dihedral driver measurement for rotation about one of the donor–acceptor bonds reveals energetic maxima located at 0° and 90° (Figure S7).^17^ The former structure, in which the **BTZ** and phenyl groups are coplanar, is energetically unfavorable due to steric hindrance between the lone pair of electrons on the **BTZ** nitrogen atom and the closest C–H bonds on the phenyl group. The second maximum corresponds to the structure in which the phenyl group is orthogonal to the **BTZ** group, disrupting the π-conjugation.

Introducing sterically bulkier *o*-phenyl (**3**) or 1-naphthyl groups (**4**) increased the torsion angle of the energy minimized structure up to approximately 60–130°, with a corresponding increase in the energy barriers to rotation (Figures S9 and S10). This can be correlated with minor hypsochromic shifts in the absorption maxima of **3** and **4**, driven by disruption of π-conjugation between the electron donor and acceptor groups. In contrast, the 2-thienyl groups of **2** present less steric hindrance with the **BTZ** group, resulting in an almost planar structure (Figure S8). This would result in improved π-conjugation across the molecule that is partly responsible for the bathochromic shift in the absorption maxima of **2** relative to **1**, although the effect of the strongly electron donating thiophene groups is also a factor.

**Figure 3:**
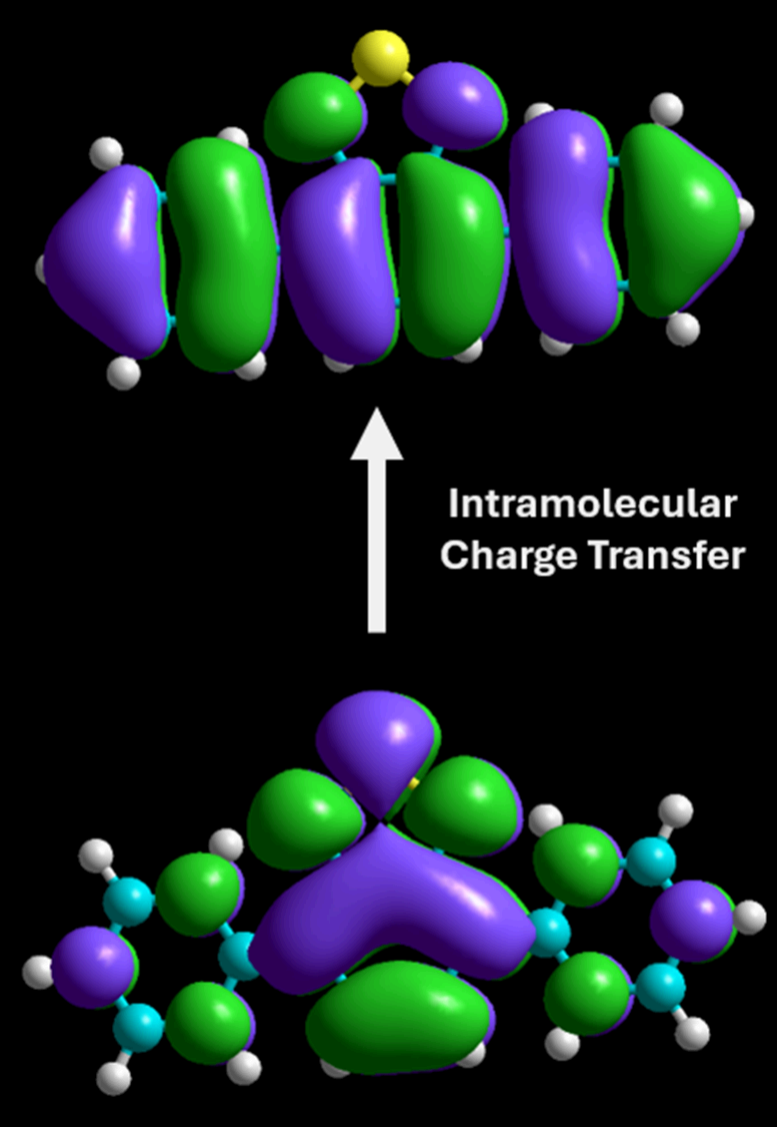
HOMO (bottom) and LUMO (top) of photocatalyst **1** calculated using the semiempirical ZINDO/s method, showing the ICT occurring following photoexcitation.

Hyperchem 8.0 was used to determine the frontier molecular orbitals (FMOs) for the photocatalysts using the semiempirical ZINDO/s method: this allowed the students to observe the intramolecular charge transfer (ICT) that occurs following photoexcitation (Table S1). The highest occupied molecule orbitals (HOMOs) for **1**–**5** are mainly located on the electron donor groups and the benzo- portion of the **BTZ** group (Figure 3). This can be contrasted with the lowest unoccupied molecular orbital (LUMOs) that is predominantly localized on the acceptor **BTZ** group. Visualizing the HOMO–LUMO orbitals for these photocatalysts was fundamental in helping the students envisage how upon absorption of light of a suitable wavelength (determined by the HOMO–LUMO gap), the electron density shifts from the electron donor to the electron acceptor blocks during the ICT step. While the rudimentary nature of these calculations reduced the computational cost, a reasonable qualitative approximation to the literature particle-orbital hole orbitals calculated using density functional theory methods (B3LYP/cc-pVTZ) were obtained.^17^

### Project Reporting

At the end of the project, the students were assessed *via* two submitted exercises. The first of these was a report written following a *pro-forma* in which the students were asked to present their written experimental data and answer questions on fundamental theory spanning physical chemistry (e.g., the Jablonski diagram), organic chemistry (e.g., redox reaction mechanisms), and computational chemistry (see the attached *pro-forma* and assessment criteria document). Reporting of the photocatalyst synthesis and characterization data was to be done using any standard publishing style: for this, the students were provided with one written example of a **BTZ** photocatalyst synthetic procedure and full spectroscopic characterization (see attached laboratory guidebook). This proforma structure allowed the students to learn how to report their data following a logical narrative in a format suitable for academic publication while relating their experience back to fundamental knowledge taught in lectures. The students were assigned overall marks based on their performance in three categories: the Mini-Project Proforma (see Supporting Information), which accounted for 50% of the total assessment and evaluated their discussion of pre-existing literature, photocatalyst synthesis, photophysical characterization, photochemical application, and computational calculations (group mark); the X (Twitter) Poster, which made up 30% of the total assessment and assessed the aesthetic appeal, the quality of discussion on the project and results, and the effectiveness of disseminating these to capture a broad audience (group mark, with peer and coordinator moderation); and Engagement, which contributed 20% of the total assessment and considered individual participation, commitment to the research project, including maintaining the lab book and adherence to lab procedures (individual mark). The overall average grades for the project over the course of 3 consecutive years were 70.0%, 73.2% and 69.1% (with respective standard deviations of 7.55, 5.84 and 4.50%) corresponding to A, A, and B using our standard metrics (A: 100–70%; B: 69–60%; C: 59–50%; D: 49–40%; E: 39–30%; F: 29–0%).

The rationale for tasking students with creating virtual posters suitable for social media platforms like X or Instagram was clearly communicated to the cohort.^20^ This approach aimed at familiarizing students with modern methods of scientific communication, which promote broader and more accessible engagement with both scientific and public audiences.^21^ Additionally, such communication methods became essential during the COVID-19 pandemic, enabling the dissemination of results in the absence of in-person conferences.^22,23^

### Student Feedback

As the main pedagogical aim of the mini project was for final year undergraduate students to acquire practical experience in organic photocatalysis, it was key to close the feedback loop and assess the student’s perception of the project. The practical experiments were carried out successfully by all groups undertaking the mini project, with yields reflected in Table 1. Students found detailed instructional videos on how to use Schlenk lines particularly helpful prior to carrying out new air sensitive reactions for the first time. Additional information about the practical use of spectrometers for photophysical measurements was also required for students who missed a significant part of their practical education due to the COVID-19 pandemic. Safety was paramount when working with LED modules: during the photoredox phase of the mini project, the emphasis was on thorough risk assessments and personal protective equipment (PPE), which students found reassuring. Most students successfully found the link between their results from computational studies and practical experiments, demonstrating an understanding that was expected from the final year undergraduates.

### Project Cost

One key consideration when designing this project was to make academic-style research financially accessible within a teaching laboratory, which is often constrained by low budgets spread across large class sizes. A cost analysis has been performed for the raw supplies needed during each of the lab exercises the students carry out (Tables S2–S6). As an example, the synthesis of photocatalyst **1** was estimated to cost $5.89 g^–1^ (£4.36 g^–1^) (Table S3). As such, assembling **BTZ** photocatalysts *via* Suzuki-Miyaura cross-coupling represents a low-cost option for teaching photocatalysis in comparison to other organic or iridium/ruthenium based photocatalysts. This was driven by the low cost of aryl boronic acids and by performing the bulk synthesis of **Br**_**2**_**BTZ** prior to the start of the mini-project, rather than directly purchasing the chemical from a commercial supplier (see Table S2).

One barrier to introducing photochemistry to the teaching lab is the cost of the light source, which can range from household white light bulbs to commercially available high power photoreactors with active cooling.^24^ Alternative light sources have been highlighted in literature that would reduce the financial barrier to photochemistry teaching projects, including the use of sunlight,^25^ fluorescent tube lights,^7^ flashlights from phones,^7^ 3D-printed photoreactors,^26^ and home-built LED modules.^6,27^ This project utilized home-built LEDs modules fabricated by mounting six 3 W LED (410–420 nm) purchased from Future Eden Ltd. onto an aluminum block that acted as a heat sink (see Figure S6). The use of low power LEDs and an aluminum heat sink meant that no cooling fans were necessary, making the LED modules easier to use and more robust. A cost analysis was performed for assembling a set of 6 LED modules: the overall cost was $80.86 (£59.83) per LED module (see Table S6). This represented the single largest initial investment of resources needed to establish this project, although the same set of LED modules is still being used after three iterations of the project.

Chem3D and Hyperchem 8.0 both require licenses to use, which cost approximately £8,000 and £6,000, respectively, per year. These were already available at Heriot-Watt University and so did not directly contribute to the cost of establishing this project, although these could be costs encountered by instructors looking to establish similar projects. Alternative software capable of running the MM2 and ZINDO/s calculations discussed in this project are available. One example is the commercial software Gaussian: this is often utilized in computational chemistry research, and so may already be available within an institution for research applications. Gaussian has already been employed in educational projects although the software requires powerful computers or servers to run efficiently.^28,29^ Alternatively, computational calculations could also be run using freeware such as ORCA,^30^ which has also seen use in educational projects.^31^

## Hazards

When in the lab, students utilized standard PPE including lab-coat, polycarbonate laboratory spectacles, and nitrile gloves. All of the synthesis, photocatalytic experiments and workups were carried out inside a fume hood. 4,7-Dibromobenzo[*c*][1,2,5]thiadiazole (**Br**_**2**_**BTZ**) is toxic. Tetrahydrofuran is flammable, is an irritant, and is carcinogenic. DCM and chloroform are both toxic and carcinogenic. Ethanol is both flammable and an irritant. Potassium carbonate is an irritant. Various boronic acids, or their pinacol esters, could be employed in this project: the five utilized here were either irritants or had no globally harmonized system (GHS) pictogram warnings. Lepidine is an irritant to the eyes, skin, and respiratory system. Cyclohexanecarboxylic acid is an irritant to the skin and damaging to the eyes. Ammonium peroxydisulfate is acutely toxic, irritating to the eyes and skin, carcinogenic, and a strong oxidant. While DMSO has no GHS symbols, it should be treated with care, as it is a skin permeation enhancer. The light source used for photocatalytic experiments (420 nm) can cause eye damage. This risk can be mediated by containing the light inside a reflective container when in use and instructing the students not to look directly at the light source when active.

## Conclusion

In summary, a mini project designed to introduce undergraduate students to photoredox catalysis has been developed. Through Suzuki-Miyaura cross-coupling, each group of students synthesized a small library of photocatalysts based on the **BTZ** electron acceptor group. By varying the aryl boronic acid used in the cross-coupling reaction, the students were able to control the photocatalysts’ properties and activity. Basic computational measurements were helpful in rationalizing these results, demonstrating the role that computational chemistry can play in photocatalyst development. By directly basing the mini project on recently published research, we were able to provide the students with a realistic, albeit condensed, experience of contemporary academic research. We envisage that this approach could be extended to a wider range of emerging photocatalysts, allowing teaching laboratories to keep pace with the evolving academic landscape. In our case, this was facilitated by utilizing home-built LED modules, which helped us to significantly reduce the cost of establishing this mini-project, making photochemistry financial accessible within teaching laboratories.

## Supporting Information Available

Mini-Project Laboratory Guidebook (PDF, DOCX)
Mini-Project Proforma and Poster Assessment Criteria (PDF, DOCX)
Notes for Instructors (PDF, DOCX)
