# Supplementary material for: A Six-Week Student-Led Project Designed to Provide Insight into Modern Photochemistry Research
Source: J Chem Educ. 2025 Mar 6;102(4):1511–7. doi: 10.1021/acs.jchemed.4c01241 (PMC11984107; doi:10.1021/acs.jchemed.4c01241)
Supplement: Supplementary file 1 — ed4c01241_si_001.pdf [file ed4c01241_si_001.pdf]

A six-week student-led project designed to provide insight into modern photochemistry research

Dominic Taylor,[a,†] Leonardo Amicosante,[a,†] Luize M. Luse,[a] Martin R. S. McCoustra,[a] Lee McMahon,[a] Scott J. Dalgarno,[a,*] and Filipe Vilela[a,*]

[†]These authors contributed equally.

[a]School of Engineering and Physical Sciences, Heriot-Watt University, Riccarton, Edinburgh, EH14 4AS, UK.

Year 4 BSc Mini-project

Synthesis and Application of 4,7-Diarylbenzo[*c*][1,2,5]thiadiazole Photocatalysts

Laboratory Guidebook

2024 - 2025

Name………………………………………………………………………………..

Introduction

Synthesis and Application of 4,7-Diarylbenzo[*c*][1,2,5]thiadiazole Photocatalysts

Keywords: Suzuki-Miyaura Cross Coupling – Photophysics – Photoredox Catalysis – Computational Chemistry

The aim of this mini-project is to provide you with an opportunity to develop skills such as designing and managing research projects, scientific collaboration and communication, and practical lab skills. In this project you will have the opportunity to synthesise a selection of photocatalysts based on the benzo[*c*][1,2,5]thiadiazole (**BTZ**) moiety. The synthesis will be *via* Suzuki-Miyaura cross coupling, which you have been taught about in your year 4 organic chemistry lectures. This mini-project will therefore give you an opportunity to complement your understanding of palladium catalysed coupling chemistry with practical experience. Once you have synthesised these photocatalysts, you will measure some photophysical properties then apply the photocatalysts to the decarboxylative alkylation of heteroarenes. As such this mini-project also complements your Year 4 lectures on photochemistry, which you may not have been exposed to in a practical environment. There is significant literature that can be investigated for further reading and understanding, some of which will be covered in the Scientific Background section of this document.

The key learning outcomes for this mini-project are:

1. to develop your practical chemistry skills by performing air-sensitive reactions.
2. to develop your understanding of organic and photochemistry.
3. to demonstrate that computational work can complement practical laboratory results.
4. to develop skills surrounding time management, teamwork, and scientific communication.

These four learning outcomes are important skills for working in actual research groups. Following completion of the mini-project, you will be asked as a group to generate a lab report using the proforma provided on Canvas. You will also be asked to prepare a Twitter poster using the example provided on Canvas as an example.

Scientific Background

In recent years, climate change stemming from anthropogenic pollution and globally depleting resources have led to discussion within the chemistry community into the development of new environmentally sustainable ways of performing chemical reactions. Among the various avenues of research, photochemistry is particularly enticing to researchers as visible light is abundant and cheap, easy to use and can provide access to transformations unattainable through other routes. In addition, our planet receives an excess of energy from the sun each day: harvesting this light to power our chemical industry could help reduce our dependence on finite fossil fuels, a vision that was realised at the end of the 19th century by Italian chemist Giacomo Ciamician.[1] As an early proponent of using light to achieve chemical transformations, Ciamician stated that:

> *"And if in a distant future the supply of coal becomes completely exhausted, civilization will not be checked by that, for life and civilization will continue as long as the sun shines."*

One class of photochemical reaction that has been extensively explored are photoredox reactions. The key mechanistic feature of this type of reaction is that the excited state photocatalyst engages in electron transfer with the substrate or sacrificial oxidants/reductants.[2] When a photocatalyst (or more specifically photoredox catalyst (PRC) when discussing this type of reaction) absorbs light, an electron will be promoted from the highest occupied molecular orbital (HOMO) to the lowest unoccupied molecular orbital (LUMO). Since this photoexcited electron is located at a higher energy level, its donation to an acceptor (A) is more favourable in comparison to the ground state. Similarly, photoexcitation of an electron leaves behind a positively charged "hole" that will more readily accept an electron from a donor (D) (Figure 1). Thus, an excited state photocatalyst is simultaneously both a stronger reducing and a stronger oxidising agent due to the electron/hole pair separation. The ability of excited state photoredox catalysts to act as both oxidisers and reductants contrasts with traditional redox chemistry, whereby a reagent can be an oxidant or a reductant, but not both.

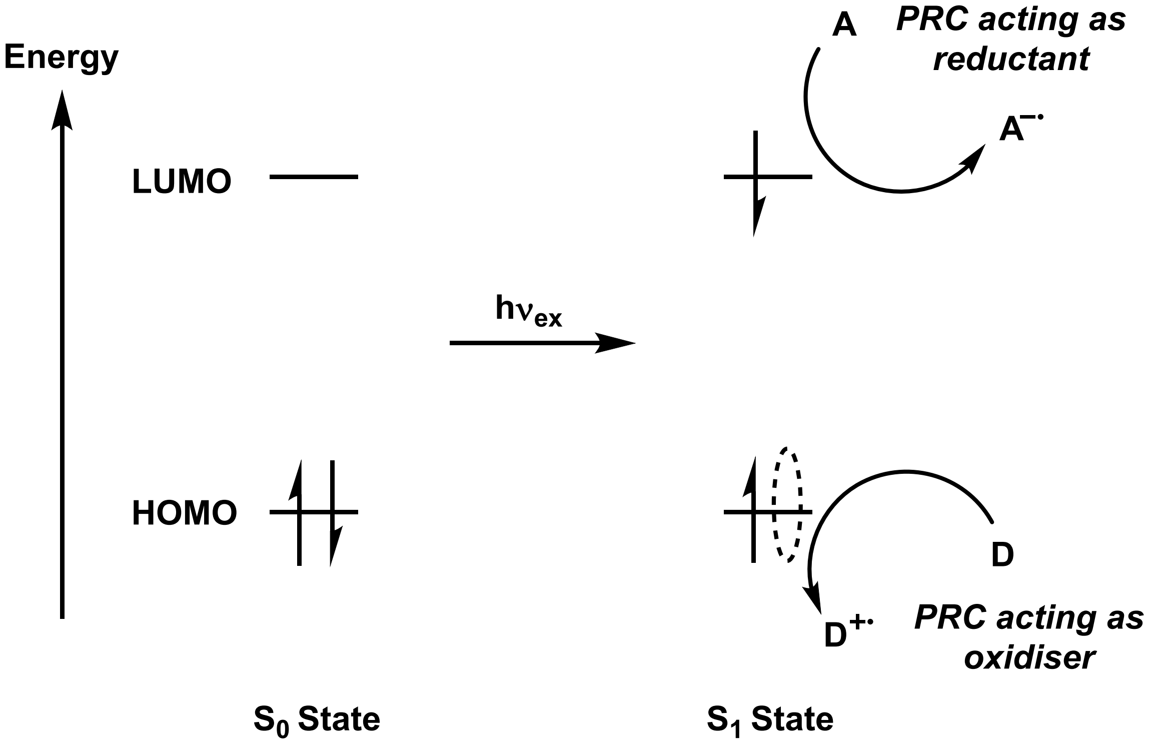

Figure 1 Photoexcitation of an electron in the HOMO to the LUMO (presumed here to be the $S_0 \rightarrow S_1$ electronic transition) by a photon with energy $h\nu_{ex}$ generates charge separation between the HOMO and LUMO. The excited state PRC can then act as a reductant by donating an electron from the LUMO or as a oxidant by accepting an electron to the HOMO.

The increase in both the oxidation and reduction power that accompanies electronic excitation of a photoredox catalyst has permitted a diverse range of novel synthetic methodologies, that may be difficult to achieve through alternative chemical routes, to be developed.[2] One such example of this is the C-H functionalisation of heteroarenes as these are abundant, naturally occurring structural motifs which have become common features in various pharmaceutical compounds, with many examples such as Papeverine, Bendamustine and Dabigatran possessing alkyl regions (Figure 2). The classical reaction to alkylate heteroarenes is the Minisci reaction, which generates alkyl radicals (R•) *via* the Ag(I) catalysed decarboxylation of carboxylic acids (RCO_2H) using persulfate anions ($S_2O_8^{2-}$) as the oxidant, which then react with the heteroarene.[3] Recent research has demonstrated that this transformation can also be achieved through the use of photoredox catalysis, which mitigates the harsh conditions presented by the Ag(I) salts.[4] However, the current methods in literature generally employ iridium based photocatalysts which are expensive and derived from resources with low natural abundance.

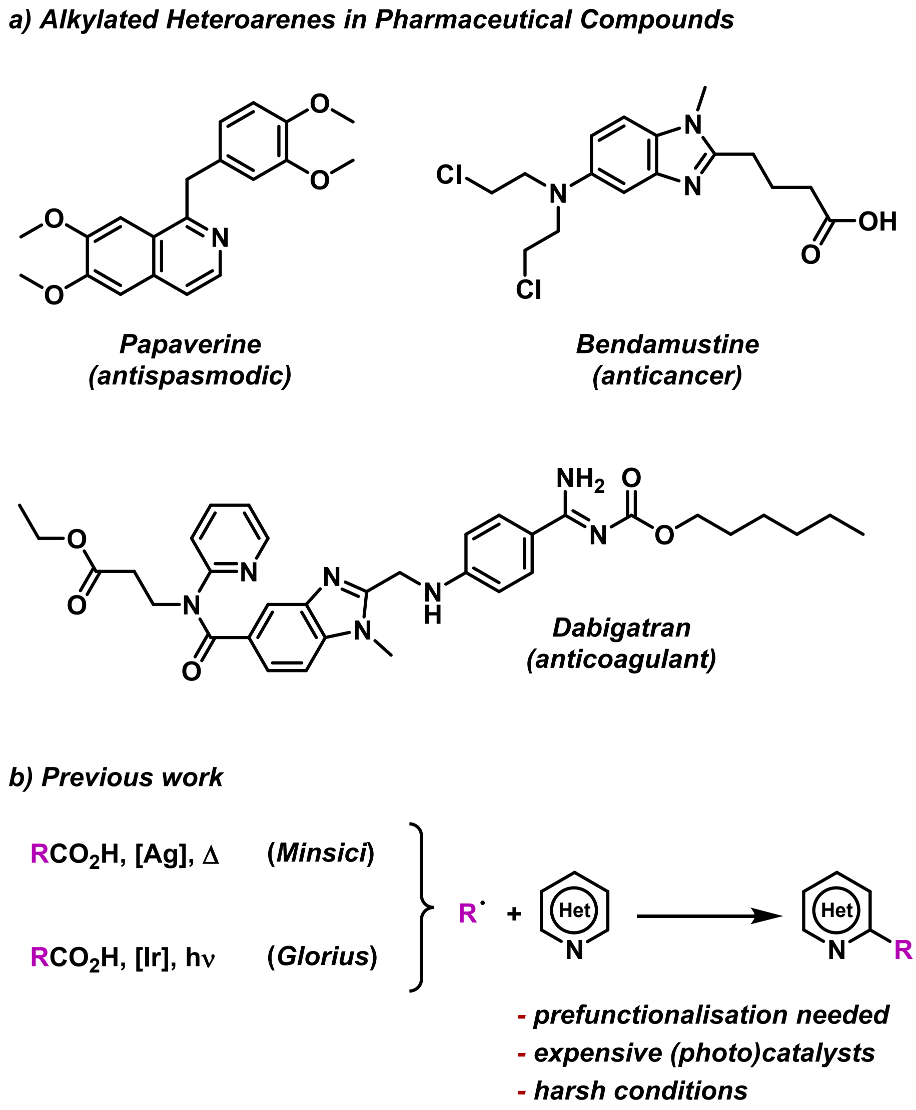

Figure 2 a) Examples of drugs featuring an alkylated heteroarene moiety. b) Examples of previous methods that have been used to alkylated heteroarenes *via* generation of alkyl radicals.

An alternative to photocatalysts based on metal-ligand complexes are entirely organic photocatalysts called organophotocatalysts.[5,6] Many different compounds have been investigated for application as photoredox organocatalysts, such as xanthenes, acridiniums, phthalocyanines and BODIPY dyes.[7,8] One class of compounds that have received less attention are photocatalysts based on the benzo[*c*][1,2,5]thiadiazole (**BTZ**) motif.[9] Photocatalysts based on these systems have, in recent years, demonstrated great versatility to catalyse a variety of photocatalytic reactions including singlet oxygen generation, the oxidation of aryl boronic acids and the photoreduction of Cr(VI) to Cr(III).[10–13] Their synthesis *via* Suzuki-Miyaura coupling from 4,7-dibromobenzo[*c*][1,2,5]thiadiazole (a cheap to make starting material) is fairly

simple, with careful selection of the boronic acid coupling partner permitting access to large libraries of photocatalysts (Scheme 1).

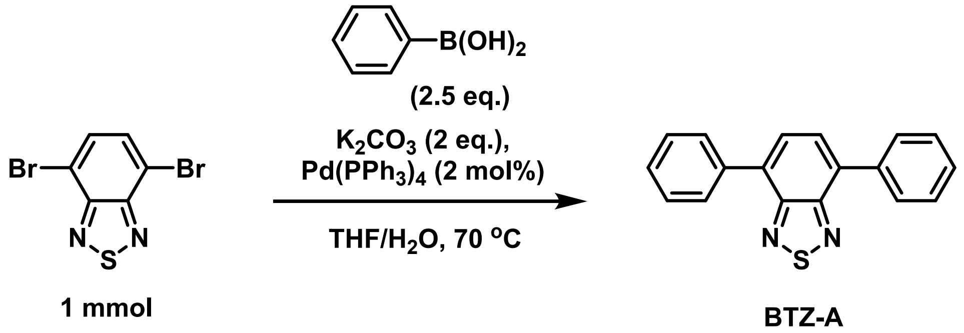

Scheme 1 Synthesis of 4,7-diarylbenzo[*c*][1,2,5]thiadiazole photocatalysts *via* Suzuki-Miyaura cross coupling, exemplified by the synthesis of 4,7-diphenylbenzo[*c*][1,2,5]thiadiazole.

In groups of three to five, you will synthesise a total of three **BTZ** based photocatalysts *via* Suzuki-Miyaura cross coupling (Scheme 1). You will then measure some photophysical properties of your photocatalyst then proceed to test your photocatalysts using the reaction shown in Scheme 2. You will also carry out computational calculations on your photocatalysts that will complement the practical experiments that you perform.

Mini-project Organisation

For this mini-project, you will be separated into the following groups:

**Group 1**	**Group 2**	**Group 3**
Group 1 Names	*Group 2 Names*	*Group 3 Names*

It is the job of the coordinator to email the responsible academic, for example, if any questions or problems arise during the project or for submitting any group work documents. Any email sent by the coordinator to the academic should have all members of the group carbon copied in.

Practical work associated with this mini-project is timetabled to run from weeks 7 to 11 of semester 1 in the second-floor teaching lab (inorganic/physical lab). The project is broken down into three sections (see timetable below).

Between 9 am and 12 pm on Mondays, Wednesdays, and Fridays of the following weeks

Semester 1 Weeks	Activity
**7**	NMR and Schlenk line Training.<br>Synthesis of photocatalysts
**8**	Full characterisation of photocatalysts<br>Including photophysical characterisation
**9**	Photocatalysis
**10**	Photocatalysis and Characterisation
In **Semester 2**, you will have 2 weeks to complete the computational studies.	

IMPORTANT

Both the physical teaching lab and the computer lab will be booked on Monday (9 am – 12 pm), Wednesday (9 am to 12 pm) and Friday (9 am to 12 pm). These sessions have been incorporated into your timetable for both semesters, **so even if you do not have lab work your group should undertake other tasks such as completing assessments.**

Synthesis and characterisation of photocatalysts – during these two weeks the groups should synthesise their photocatalysts. This will be done *via* Suzuki-Miyaura cross coupling. **Before you arrive at the lab, it is highly advised that you watch the video on Canvas on how to carry out this reaction and how to properly use a Schlenk line**. A demonstrator will provide a demonstration of how to correctly use a Schlenk line for the Suzuki-Miyaura cross coupling during the first lab session.

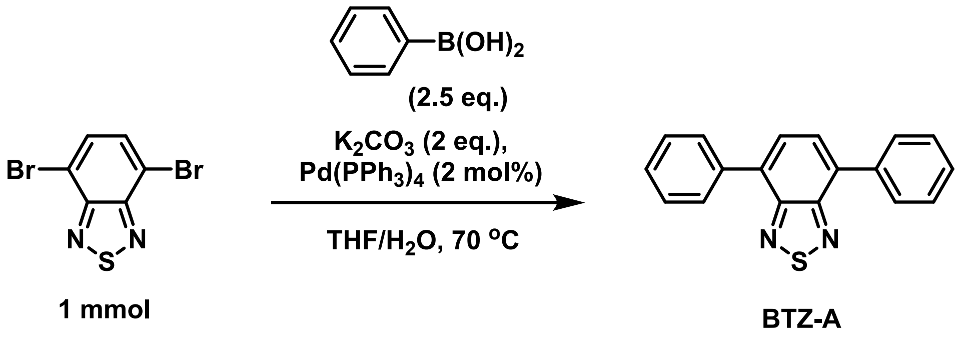

Each group will synthesise three photocatalysts. All groups should synthesise both **BTZ A** and **BTZ B**.

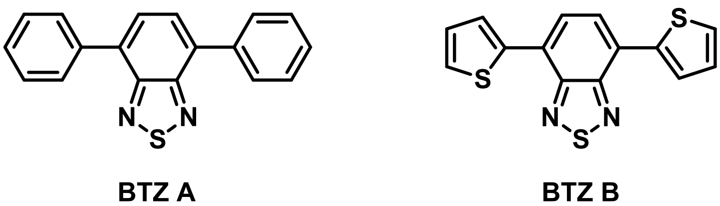

Each group should also synthesise the photocatalysts from the series shown below that corresponds to their group number.

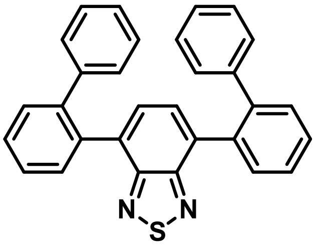

BTZ Group 1

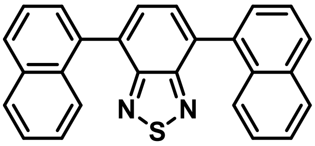

BTZ Group 2

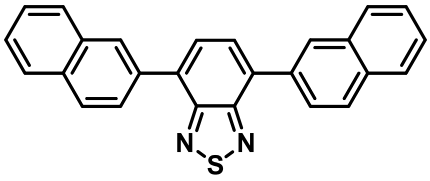

BTZ Group 3

The synthesis of these photocatalysts typically takes between 16 and 72 hours. To determine if your reaction is complete, you should run a crude ^{1}H NMR spectra. This is done by withdrawing a sample from your reaction mixture using a glass pipette, removing the solvent under reduced pressure, and dissolving the residue in $CDCl_3$ for analysis by ^{1}H NMR. The spectra you will obtain will contain a lot of impurities but should be clean enough to observe if starting material is present. Do not remove the reaction before confirming that the reaction is done – it is extremely difficult to purify if you remove it early.

Once synthesised, full structural characterisation should be carried out and reported in a proper scientific manner (see Appendix 1). This includes carrying out ^{1}H and ^{13}C NMR spectroscopy, any relevant multinuclear NMR spectra, IR spectroscopy and UV-Vis absorption spectra. You will also be given the high-resolution mass spectra data for your compound (you can report this as if you recorded the data).

Photophysical Characterisation and Application - Once you have made your photocatalyst, you should study its photophysical properties and then validate its performance using a test photoredox reaction. The photophysical characterisation that should be carried out includes:

- Recording UV-Vis absorption spectra in chloroform for all photocatalysts.
- Recording emission spectra for all photocatalysts in chloroform.
- Recording UV-Vis absorption and emission spectra in alternative solvents. Some useful examples/information on solvatochromism is presented in references 14–18.
- Determining molar attenuation coefficients for your photocatalysts with associated error.

The physical teaching lab has two UV-Vis absorption spectrometers and one fluorescence spectrometer – the group coordinators of the groups should liaise with each other to enable fair access to all of the instruments.

Once characterised, you will apply your photocatalysts to the C-H functionalisation of lepidine using cyclohexane carboxylic acid (shown below).[4,19] Each group will be given an LED module with which to carry out all the relevant photocatalytic experiments. **Before you arrive at the lab, it is highly advised that you watch the video on Canvas on how to carry out this reaction**. You should first optimise your reaction conditions by changing conditions such as solvent, oxidant, concentration of photocatalyst and time in such a way to favour higher reaction conversions. When optimised conditions for one photocatalyst have been established you should compare it to the performance of your two other photocatalysts. You should also perform any relevant control experiments.

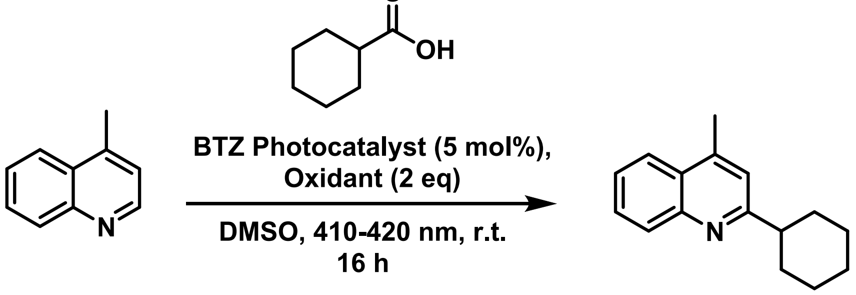

Computational Calculations - To complement your photophysical characterisation of your series of photocatalysts, you are also tasked with performing computational calculations using Chem3D and Hyperchem. Chem3D can be downloaded to your own PC or laptop as part of the ChemDraw suite, freely available from the university. Both Chem3D and Hyperchem are available on computers in the chemistry computer lab, which has been booked for your use. Using Chem3D, you should first create energy minimised optimised structures for each of your photocatalysts then determine the energy barrier for rotation around the bond connecting the **BTZ** acceptor and the donor aryl group. Following this, create energy minimised structures in Hyperchem. Try a few different molecular mechanics force fields to find out which one works best for your molecules (*e.g.,* MM+). The energy minimised structures obtained in Hyperchem should resemble as closely as possible the structures you obtained using Chem3D. A conformational search in Hyperchem can be used to alter the torsion angles if necessary. Then run a single point calculation using the ZINDO/1 method and determine the energies and shapes of the HOMO and LUMO.

Mini-project Assessment and Feedback on Activities

The mark that you will be awarded for this project will comprise of several components that are assessed either on your individual performance or as part of a group.

Assessment	% Grade
**Experiment Proforma and Electronic Supporting Information (Group Mark)**<br>*Marks awarded for discussion of pre-existing literature, photocatalyst synthesis, photophysical characterisation, photochemical application, and computational calculations (85 marks). An electronic supporting information document containing all of your characterisation spectra should also be submitted (15 marks). This assessment will be marked by the mini-project coordinator.*	50%
**X (Twitter) Poster (Group Mark)**<br>*Marks awarded for aesthetic appeal of the poster and discussion of the experimental and computational results. This assessment will be marked by the other groups with moderation from the mini-project coordinator.*	30%
**Engagement (Individual Mark)**<br>*Marks awarded for participation in group activities, commitment to the research project including the lab book and overall adherence to lab procedures. This mark will be awarded by the mini-project coordinator.*	20%

IMPORTANT

Although the marks you receive for the experiment proforma and X (Twitter) poster are group mark, any individual that does not contribute to the final submitted material will not receive that group mark and instead receive revised marks.

The experiment proforma and peer assessment form can both be found on Canvas, along with an example of a X (Twitter) poster. Once your submitted material has been graded, you will receive feedback through an uploaded document on Canvas.

IMPORTANT

Dates for submission of graded work:

Assessment	Date Due
Experiment Proforma	**5pm Friday, Semester 2 Week 4**
X (Twitter) Poster	**5pm Friday, Semester 2 Week 5**
Peer Assessment	**5pm Friday, Semester 2 Week 6**

Submission of work after the deadlines will be penalised in accordance with the university's rules. This is an automatic deduction as determined by the university's rules from the final mark unless your group/individual has mitigating circumstances that are upheld.

References

1 G. Ciamician, *Science (80-.).*, 1912, **36**, 385–394.

2 N. A. Romero and D. A. Nicewicz, *Chem. Rev.*, 2016, **116**, 10075–10166.

3 F. Minisci, R. Bernardi, F. Bertini, R. Galli and M. Perchinummo, *Tetrahedron*, 1971, **27**, 3575–3579.

4 R. A. Garza-Sanchez, A. Tlahuext-Aca, G. Tavakoli and F. Glorius, *ACS Catal.*, 2017, **7**, 4057–4061.

5 C. K. Prier, D. A. Rankic and D. W. C. MacMillan, *Chem. Rev.*, 2013, **113**, 5322–5363.

6 M. H. Shaw, J. Twilton and D. W. C. MacMillan, *J. Org. Chem.*, 2016, **81**, 6898–6926.

7 M. C. DeRosa and R. J. Crutchley, *Coord. Chem. Rev.*, 2002, **233–234**, 351–371.

8 A. Kamkaew, S. H. Lim, H. B. Lee, L. V. Kiew, L. Y. Chung and K. Burgess, *Chem. Soc. Rev.*, 2013, **42**, 77–88.

9 B. A. D. Neto, A. A. M. Lapis, E. N. Da Silva Júnior and J. Dupont, *European J. Org. Chem.*, 2013, 228–255.

10 K. Zhang, D. Kopetzki, P. H. Seeberger, M. Antonietti and F. Vilela, *Angew. Chemie Int. Ed.*, 2013, **52**, 1432–1436.

11 B. C. Ma, S. Ghasimi, K. Landfester, F. Vilela and K. A. I. Zhang, *J. Mater. Chem. A*, 2015, 3, 16064–16071.

12 J. M. Tobin, T. J. D. McCabe, A. W. Prentice, S. Holzer, G. O. Lloyd, M. J. Paterson, V. Arrighi, P. A. G. Cormack and F. Vilela, *ACS Catal.*, 2017, **7**, 4602–4612.

13 S. Ghasimi, K. Landfester and K. A. I. Zhang, *ChemCatChem*, 2016, **8**, 694–698.

14 M. Chen, H. Nie, B. Song, L. Li, J. Z. Sun, A. Qin and B. Z. Tang, *J. Mater. Chem. C*, 2016, **4**, 2901–2908.

15 S. Chen, X. Li and L. Song, *RSC Adv.*, 2017, **7**, 29854–29859.

16 D. Jana and S. Jana, *ACS Omega*, 2020, **5**, 9944–9956.

17 C. Reichardt, *Angew. Chemie Int. Ed. English*, 1965, **4**, 29–40.

18 C. Reichardt, *Chem. Rev.*, 1994, **94**, 2319–2358.

19 D. Taylor, T. Malcomson, A. Zhakeyev, S. Cheng, G. M. Rosair, J. Marques-Hueso, Z. Xu, M. J. Paterson, S. J. Dalgarno and F. Vilela, *Org. Chem. Front.*, 2022, **9**, 5473–5484.

Appendix 1

How to properly write a scientific procedure and report spectroscopic characterisation

When a chemist reports the synthesis of a compound, they generally word the procedure and spectroscopic characterisation in a particular manner. There is no set style, but procedures tend to be written impassively in the past tense. For example, instead of saying "We added THF to the reaction" instead say "THF was added to the reaction". The use of personal pronouns like "I", "we" *etc.* should be avoided in procedures as chemists should be objective with their reporting. The level of detail should be sufficient that another student in your year should be able to implement the procedure. You should also be concise as possible: omit any information which does not need to be specified to accurately replicate the procedure.

Up until now, you will have most likely reported NMR data using a table of your inferences. It would be assumed that a trained chemist would understand which peaks correspond to which atoms so instead you just report the signal, its integration, the DEPT spectra, its multiplicity and coupling constant if applicable:

- Reports of ^{1}H NMR spectra should specify the chemical shift (to two decimal places) from most downfield to most upfield peak. In brackets the integration of the peak, the multiplicities and the coupling constants should be specified if applicable. Coupling constants should be reported to one decimal place.
- Reporting ^{13}C NMR require the chemical shift (to one decimal place) then the inference from the DEPT spectra in brackets ($C/CH/CH_2/CH_3$).
- Multinuclear NMR should be written in a similar manner to ^{1}H NMR spectroscopic data.
- UV-Vis absorption spectra should specify the solvent used and the maximum wavelength(s). Generally, you would only be concerned with maxima above 300 nm so you do not need to report any maxima in the far UV region.
- IR spectra should specify the wavenumber of the signal in cm^{-1}, the type of bond vibrating, the type of vibration (stretching, bending) and its strength (strong, weak).
- HRMS should specify the theoretical mass corresponding to the molecular ion peak and the observed peak accurate to four decimal places.
- In addition to writing the characterisation in the main report, you should also add annotated spectra to an electronic supplementary information (ESI) document. This is to show that you have synthesised the molecule you have claimed to have synthesised.

Example of Lab Book entry

GR1-001[a] Date

Note any absences from your group

[a]Note that GR1 is your group number and 001 is the experiment number.

EXPERIMENT TITLE

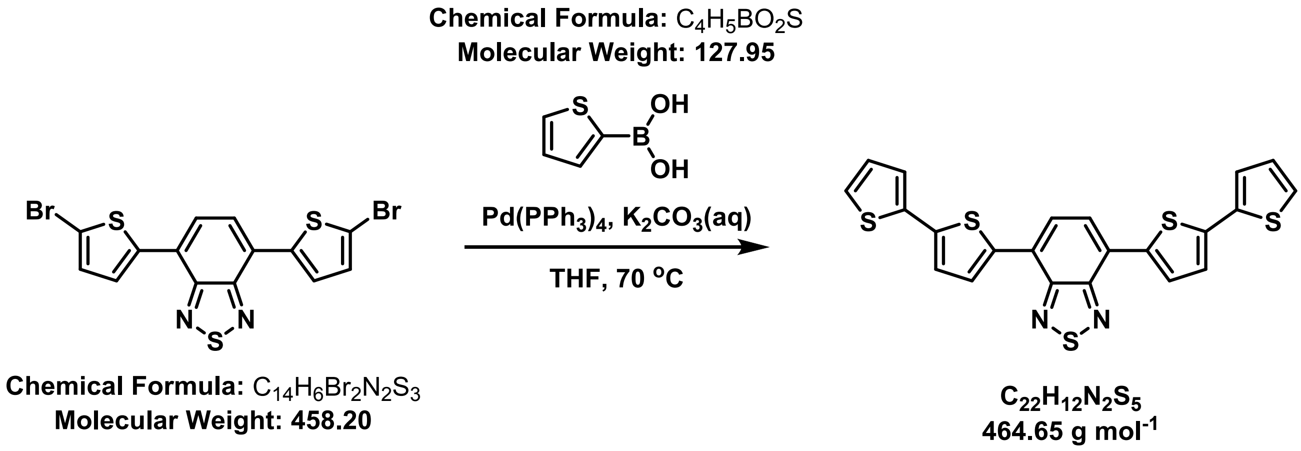

Table of Reagents

Chemical	Mw (g.mol^{-1})	mmol	Eq.	m/V	Supplier
4,7-*bis*(5-bromothiophen-2-yl)benzo[*c*][1,2,5]thiadiazole	458.20	0.5	1	229 mg	Sigma-Aldrich
Thiophene-2-boronic acid	127.95	1.25	2.5	160 mg	Fluorochem
$Pd(PPh_3)_4$	1155.59	0.01	2 mol%	12 mg	Fluorochem
Potassium Carbonate	138.21	1	2	138 mg	TCI
THF	-	-	-	40 mL	Fisher
Deionised Water	-	-	-	5 mL	From Lab

A dry 2-neck flask was charged with 4,7-*bis*(5-bromothiophen-2-yl)benzo[*c*][1,2,5]thiadiazole (229 mg, 0.5 mmol), thiophene-2-boronic acid (160 mg, 1.25 mmol), potassium carbonate (138 mg, 1.0 mmol) and $Pd(PPh_3)_4$ (12 mg, 0.01 mmol). The flask was connected to a Schlenk line then evacuated and backfilled with nitrogen three times. Degassed THF (40 mL) and degassed

deionised water (5 mL) were added, and the reaction heated to 70 °C overnight. Following this time, the mixture was allowed to cool to room temperature, poured onto deionised water and extracted with DCM (3 x 25 mL). The combined organic phases were washed with deionised water (50 mL), dried over $MgSO_4$ and then the solvent removed under reduced pressure. The crude product was then washed with hot ethanol to give a metallic purple coloured powder (160 mg, 69%).

Make note of any observations such as changes in colour

Make note of any mistakes/problems during the synthesis

Take photographs

Add any relevant reference here.

Once you have completed the analysis add the following:

^{1}H NMR ($CDCl_3$, 300 MHz, 25.0 °C) δ_H 8.06 (d, J = 4.0 Hz, 2 *H*), 7.87 (s, 2 *H*), 7.31 (dd, 2 *H*, J = 3.6, 1.1 Hz), 7.28 (d, J = 4.0 Hz, 2 *H*), 7.28 (m, 2 *H*), 7.07 (dd, J = 5.1, 3.6 Hz, 2 *H*). **^{13}C NMR** ($CDCl_3$, 75.5 MHz, 25.0 °C) δ_C 139.0 (C), 138.1 (C), 137.3 (C), 128.3 (C), 128.0 (C), 125.6 (C), 125.2 (CH), 124.9 (CH), 124.6 (CH), 124.1 (CH). **UV-Vis** ($CHCl_3$) λ_{max} (nm) 505. **IR** $\bar{\nu}$ (cm^{-1}) 3100 (w, C-H str.). **HRMS** (ES+, MeCN) m/z = 454.9651 $[M+H]^+$, calc. 464.9682.

Add concluding remarks
