# Supplementary material for: A Six-Week Student-Led Project Designed to Provide Insight into Modern Photochemistry Research
Source: J Chem Educ. 2025 Mar 6;102(4):1511–7. doi: 10.1021/acs.jchemed.4c01241 (PMC11984107; doi:10.1021/acs.jchemed.4c01241)
Supplement: Supplementary file 3 — ed4c01241_si_003.pdf [file ed4c01241_si_003.pdf]

A six-week student-led project designed to provide insight into modern photochemistry research

Dominic Taylor,[a,†] Leonardo Amicosante,[a,†] Luize M. Luse,[a] Martin R. S. McCoustra,[a] Lee McMahon,[a] Scott J. Dalgarno,[a,*] and Filipe Vilela[a,*]

[†]These authors contributed equally.

[a]School of Engineering and Physical Sciences, Heriot-Watt University, Riccarton, Edinburgh, EH14 4AS, UK.

GROUP NUMBER – **DATE -**

GROUP MEMBERS -

PROJECT TITLE -

Introduction:

Please write a short introduction (300 words max.) describing what is photochemistry/photoredox catalysis and why it is important in today's society. You should make use of figures and references where applicable (follow any of the reference systems used by RSC, ACS, Wiley, Nature or MDPI publishers) **(10 marks available)**.

Aims:

Briefly summarise the aims of this mini-project (max. 50 words) **(2 marks available)**.

Section 1 – Synthesis and Characterisation of Photocatalysts

General Reaction Scheme:

Insert a general reaction scheme for the Suzuki-Miyuara cross coupling reaction you performed **(2 marks available).**

General Experimental Procedure:

Write a brief general procedure to describe the synthesis of all three of your photocatalysts. You should also note where the synthesis of any of the photocatalyst deviated from the general procedure. There is no need to include any table of reagents **(3 marks available)**.

Results and characterisation:

Tabulate the yields that you obtained of each photocatalyst. For each photocatalyst, report the ^{1}H NMR, ^{13}C NMR, UV-Vis, UR and HRMS spectral data using proper notation. You should also include each of these spectra in the electronic supporting information (do not include them in this document) to demonstrate that you have made the photocatalysts **(10 marks available)**.

Section 2 – Photophysical Characterisation and Application

General Reaction Scheme:

Insert a general reaction scheme for Minisci-type photocatalytic reaction you performed **(2 marks available)**.

General Experimental Procedure:

Write a brief general procedure to describe your Minisci-type photocatalytic reaction. Clearly indicate on an NMR spectra which peaks you would utilise to determine the conversion by NMR **(4 marks available)**.

Photocatalytic Results:

Tabulate the NMR yields that you obtained under different conditions. Discuss your results and provide a rationalisation for the difference in performance between different conditions and photocatalysts **(6 marks available)**.

Photophysical Results:

Tabulate the photophysical data that you recorded for your photocatalysts (add UV-Vis absorption and emission spectra for each photocatalyst to the ESI). Explain how and why the three different photocatalysts you synthesised exhibited different absorption and emission profiles. You should also report the Stoke's shift and optical energy gap, as well as summarise the photocatalysts solvatochromic properties **(6 marks available)**.

Section 3 – Computational Chemistry

Chem3D Minimised Energy:

For each of your photocatalysts, report and discuss the MM2 optimised structure and its energy. You should also report and discuss the conformational energy plots **(5 marks available).**

Hyperchem Orbital Calculations:

For each of your photocatalysts, report and discuss the structure of the HOMO and LUMO as figures. In addition, tabulate the energies of the HOMO and LUMO for the optimised structure and calculate the theoretical band gap **(5 marks available)**.

Section 4 – Discussion of Results

In this section, you should answer each of the questions below (the chemistry discipline is shown in brackets). Where relevant, include reference to your results from this project.

Question (Physical): Explain the difference between the terms photosensitiser and photoredox catalyst **(2 marks)**.

Question (Physical): Draw a simple Jablonski diagram demonstrating the different photophysical processes that can occur with your photocatalysts **(4 marks)**.

Question (Physical): Explain the origin of the solvatochromic effects observed with **BTZ** photocatalysts and why these effects are more significant in the emission spectra of your photocatalysts compared to the absorption spectra and more pronounced with polar solvents **(2 marks)**.

Question (Organic): State some of the different types of chemical modifications that could result in a photocatalysts wavelength of maximum absorption shifting to lower energies. For what reason(s) might such a shift in the absorption be desired **(3 marks)**.

Question (Inorganic): Draw a mechanism for the Suzuki-Miyuara cross coupling that you performed. Include the electron count of the palladium metal center and its oxidation state at each different stage of the mechanism **(7 marks)**.

Question (Inorganic): When you go to set up the synthesise of your groups unique photocatalyst you discover that the boronic acid you need has been finished. Provide two alternate pathways from commonly available starting materials to make your groups third and unique photocatalyst that avoid using Suzuki-Miyuara coupling whilst still employing metal based catalysts/reagents. Rationalise which of these two routes you would take if all of the chemicals were available to you **(3 marks)**.

Question (Organic): The addition of (2,2,6,6-tetramethylpiperidin-1-yl)oxidanyl (also called TEMPO) suppressed the photoredox reaction, resulting in 0% yield. Explain what information about the reaction mechanism this reveals **(2 marks)**.

Question (Organic): What are control experiments and why must they be carried out when developing photocatalytic reactions. State four different control experiments that could have been performed for the test Minisci reaction **(2 marks)**.

Question (Organic): Rationalise which of the carboxylic acids shown below you would expect to perform best and worst in the test photoredox reaction **(3 marks)**.

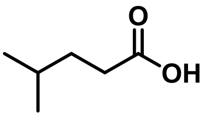

C1

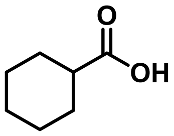

C2

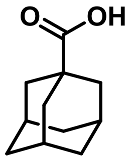

C3

Question (Physical): Using the orbital diagrams you calculated for any of your photocatalysts, explain the movement of electrons within your molecule during and after absorption. What is the name given to the mechanism of this electron movement **(2 marks)**.

Assessment Criteria for Supporting Information

Breakdown of marks **(15 marks available)**:

- UV-Vis spectra included (3 x 1 mark).
- Emission spectra included (3 x 1 mark).
- Proton and Carbon NMR spectra included (6 x 1 mark).
- IR spectra included (3 x 1 mark).
- Deduct ½ mark for mistakes on the spectra including:
    - Unlabelled axes
    - Peaks not labelled
    - Integrals not shown where appropriate
    - Solvent other than water, CDCl3 or acetone present (these are all in $CDCl_3$) present
- Deduct 1 mark for mistakes including:
    - Difficult to see figures
    - Photocatalysts not purified properly

Assessment Criteria for Scientific Mini-Project: X (Twitter) Poster

1. Aesthetic Appeal (20%)

- **Visual Design (10%)**: Marks awarded for organization, use of color, professional appearance, and overall layout.
- **Clarity and Readability (10%)**: Evaluation of text legibility, logical formatting, and effective use of space.

2. Scientific Content and Communication (40%)

- **Accuracy of Scientific Information (10%)**: Assessment of the correctness and reliability of concepts, methods, and findings presented.
- **Experimental Highlights (10%)**: Evaluation of how well key results, methods, and findings are summarized and communicated.
- **Data Presentation (10%)**: Marks for the clarity, accuracy, and appropriateness of graphs, tables, and other visualizations.
- **Context and Relevance (10%)**: Focus on framing the research within a broader scientific or societal context.

3. Engagement and Accessibility (20%)

- **Target Audience Adaptation (10%)**: How effectively the poster communicates to both a specialist and non-specialist audience.
- **Impact and Messaging (10%)**: Assessment of the strength and clarity of the core message or takeaway.

4. Collaboration and Group Work (10%)

- **Team Effort and Coordination (5%)**: Evaluation of the balance of contributions and cohesiveness in the final product.
- **Responsiveness to Feedback (5%)**: Marks for evidence of iterative improvement and incorporation of suggestions.

5. Innovation and Creativity (10%)

- **Novelty in Presentation (5%)**: Marks for originality and creativity in the presentation style or approach.
- **Integration of Modern Tools (5%)**: Use of advanced tools for visuals or other elements that enhance the presentation.

6. Adherence to Scientific Standards (10%)

- **Citation and Referencing (5%)**: Proper attribution of sources, data, and frameworks.
- **Scientific Integrity (5%)**: Transparent and unbiased presentation of methods, data, and conclusions.
