# Supplementary material for: A Six-Week Student-Led Project Designed to Provide Insight into Modern Photochemistry Research
Source: J Chem Educ. 2025 Mar 6;102(4):1511–7. doi: 10.1021/acs.jchemed.4c01241 (PMC11984107; doi:10.1021/acs.jchemed.4c01241)
Supplement: Supplementary file 5 — ed4c01241_si_005.pdf [file ed4c01241_si_005.pdf]

A six-week student-led project designed to provide insight into modern photochemistry research

Electronic Supplementary Information and Notes for Instructors

Dominic Taylor,[a,†] Leonardo Amicosante,[a,†] Luize M. Luse,[a] Martin R. S. McCoustra,[a] Lee McMahon,[a] Scott. J. Dalgarno[a,*] and Filipe Vilela[a,*]

[a]School of Engineering and Physical Sciences, Heriot-Watt University, Riccarton, Edinburgh, EH14 4AS, UK.

[†]These authors contributed equally.

Table of Contents

**1.**	**Experimental Procedures**	**2**
	**1.1 Synthesis of Starting Chemicals**	**2**
	**1.2 Synthesis and Characterisation of Photocatalysts**	**3**
	**1.3 Example Photoredox Procedure**	**8**
	**1.4 Computational Methods**	**10**
**2.**	**Cost Analysis**	**14**
**3.**	**^{1}H NMR Spectra of Pure Starting Materials and Products**	**17**
**4.**	**References**	**21**

1. Experimental Procedures

1.1 Synthesis of Starting Chemicals

4,7-Dibromobenzo[*c*][1,2,5]thiadiazole (Br_2BTZ)[1]

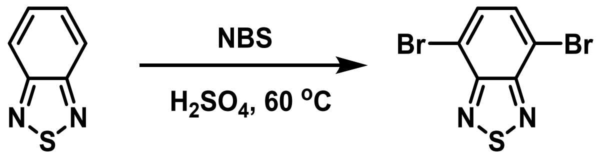

A solution of benzo[*c*][1,2,5]thiadiazole (2.5 g, 18.4 mmol) and *N*-bromosuccinimide (NBS) (6.877 g, 38.64 mmol) in concentrated sulfuric acid (25 mL) was heated to 60 °C for 4 hours. The reaction mixture was allowed to cool to room temperature, then slowly poured directly onto an ice-water mixture. The precipitate that formed was filtered, washed with water until the filtrate tested neutral on pH paper, then dried under vacuum to give a white powder (4.524 g, 85%). **^{1}H NMR** ($CDCl_3$, 300 MHz) δ_H 7.73 (s, 2 *H*). **^{13}C NMR** ($CDCl_3$, 75.5 MHz, 25.0 °C) δ_C 153.0 (C), 132.3 (CH), 113.9 (C).

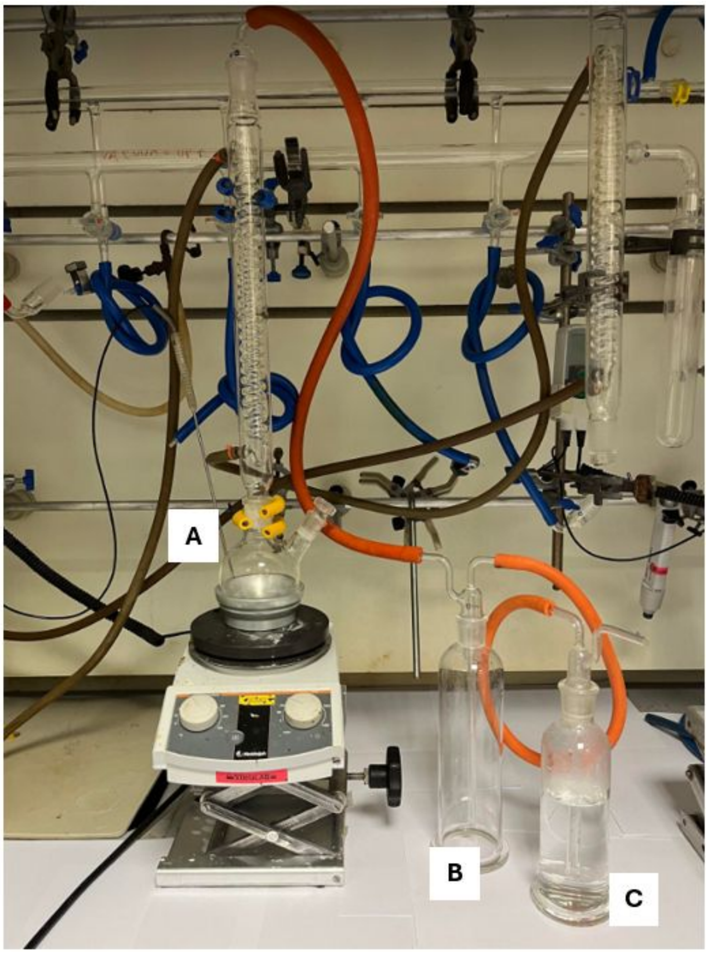

Figure S1 Experimental set up used to synthesise **Br_2BTZ**.

NOTES TO INSTRUCTORS

- For safety, the reflux apparatus (A) was connected to a set of two Dreschel gas washing bottles to neutralise any escaping HBr vapor. The first washing bottle (B) was empty while the second contained a concentrated NaOH (aq) solution (C). The purpose of leaving the first bubbler empty is in case of an unexpected temperature drop in the reaction, NaOH solution will not be pulled into the reaction mixture.
- The ^{1}H NMR spectra of the crude product following reaction may show traces of the partially brominated product. This impurity can be removed by recrystallisation from hot ethanol or hot chloroform.
- **Br_2BTZ** can also be synthesised by reaction of **BTZ** with bromine in hydrobromic acid or is commercially available.[2] If Br_2/HBr is used as the bromination conditions then the same neutralisation set up shown in Figure S1 can be used.

1.2 Synthesis and Characterisation of Photocatalysts

General Procedure for Synthesis of Photocatalysts

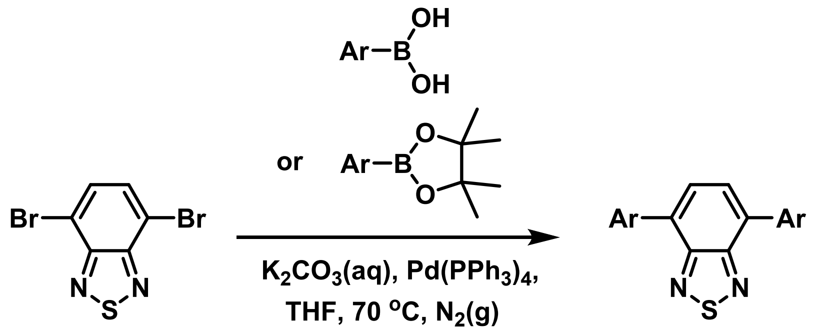

A dry 2-neck flask containing **Br_2BTZ** (1.0 mmol), aryl boronic acid (2.5 mmol), potassium carbonate (2.0 mmol) and $Pd(PPh_3)_4$ (5 mol%) was connected to a Schlenk line then evacuated and back-filled with nitrogen three times. Degassed THF (40 mL) and degassed deionised water (5 mL) were added and the reaction heated to 70 °C overnight under an atmosphere of nitrogen. After the mixture cooled to room temperature, it was poured onto water and extracted with DCM (3 x 25 mL). The combined organic phases were washed with deionised water (50 mL), dried over $MgSO_4$ and the solvent removed under reduced pressure. The crude product was purified by either recrystallisation or washing the powder with hot ethanol.

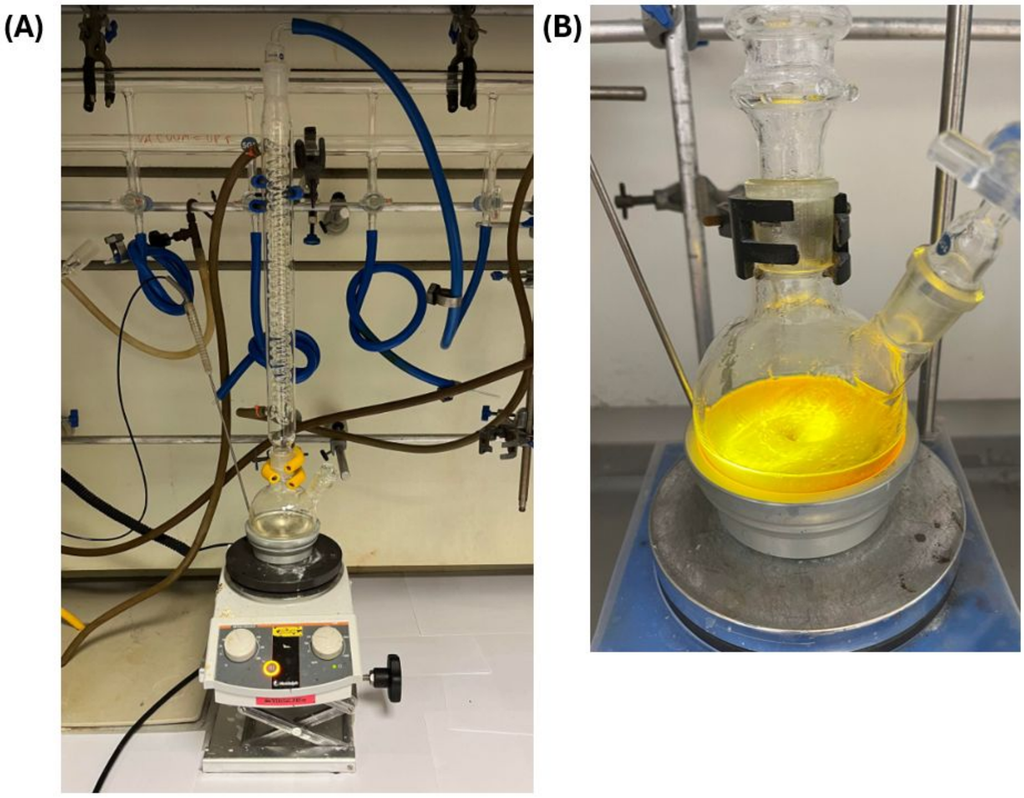

Figure S2 (A) Experimental set up for the Suzuki-Miyaura cross coupling reaction. (B) An in-progress Suzuki-Miyaura cross coupling reaction exhibiting fluorescence under UV illumination, due to the presence of a **BTZ** photocatalyst (**2**).

NOTES TO INSTRUCTORS

- The synthesis of **BTZ** photocatalysts *via* Suzuki-Miyaura cross coupling was most effective with electron rich or electron neutral aryl boronic acids. Boronic acids bearing electron withdrawing groups are not suitable as the rate of the Suzuki-Miyaura cross coupling reaction is very slow.
- An instructional video was made available to the undergraduate students demonstrating how to carry out a Suzuki-Miyaura cross coupling reaction. Supporting videos can be found in the VilelaLab channel on Youtube.
  Suzuki Miyaura cross-coupling reaction:
  https://www.youtube.com/watch?v=EyilEmKbux4
  Photochemical Minisci reaction:
  https://www.youtube.com/watch?v=30Z7LQcK3yI
- We have found that using diaphragm pumps to evacuate and back-fill the reaction flask is sufficient to achieve conditions inert enough for these Suzuki-Miyaura cross-coupling reactions. This avoids the use of oil pumps, which are more susceptible to solvent damage and require liquid nitrogen handling for solvent traps.
- $Pd(PPh_3)_4$ was selected as the catalyst over generating an *in situ* catalyst due to its air sensitivity, providing an opportunity to demonstrate to the students the need for proper treatment of

chemicals to ensure longevity. This learning objective is somewhat facilitated by the bright yellow colour of the catalyst, which fades to orange upon prolonged exposure to air, providing a visual indicator of purity. After each use, the students were instructed to seal the flask containing the $Pd(PPh_3)_4$ with a septum, then evacuate and back-fill the flask with nitrogen gas to purge the system of oxygen before storage in a freezer.

- It is advised that the students perform a crude ^{1}H NMR spectra on their reactions to determine if the reaction was complete. This is facilitated by the presence of identifiable signals corresponding to the **BTZ** group of **Br$_2$BTZ**, the monocoupled intermediate and the dicoupled target material (see Figure S3). Separation of these three intermediates requires challenging silica gel column chromatography.
- If the reaction looks as if it has stalled due to the ingress of oxygen, the reaction can be cooled, degassed by bubbling the solution with nitrogen gas then adding more $Pd(PPh_3)_4$.
- Photocatalysts **1** – **5** could all be purified by either recrystallisation or washing with hot ethanol. If the introduction of any target **BTZ** photocatalysts required chromatographic resolution, this would be facilitated by their intense fluoroscence under long-wave UV illumination, making them easily visible while loaded onto a silica gel column.

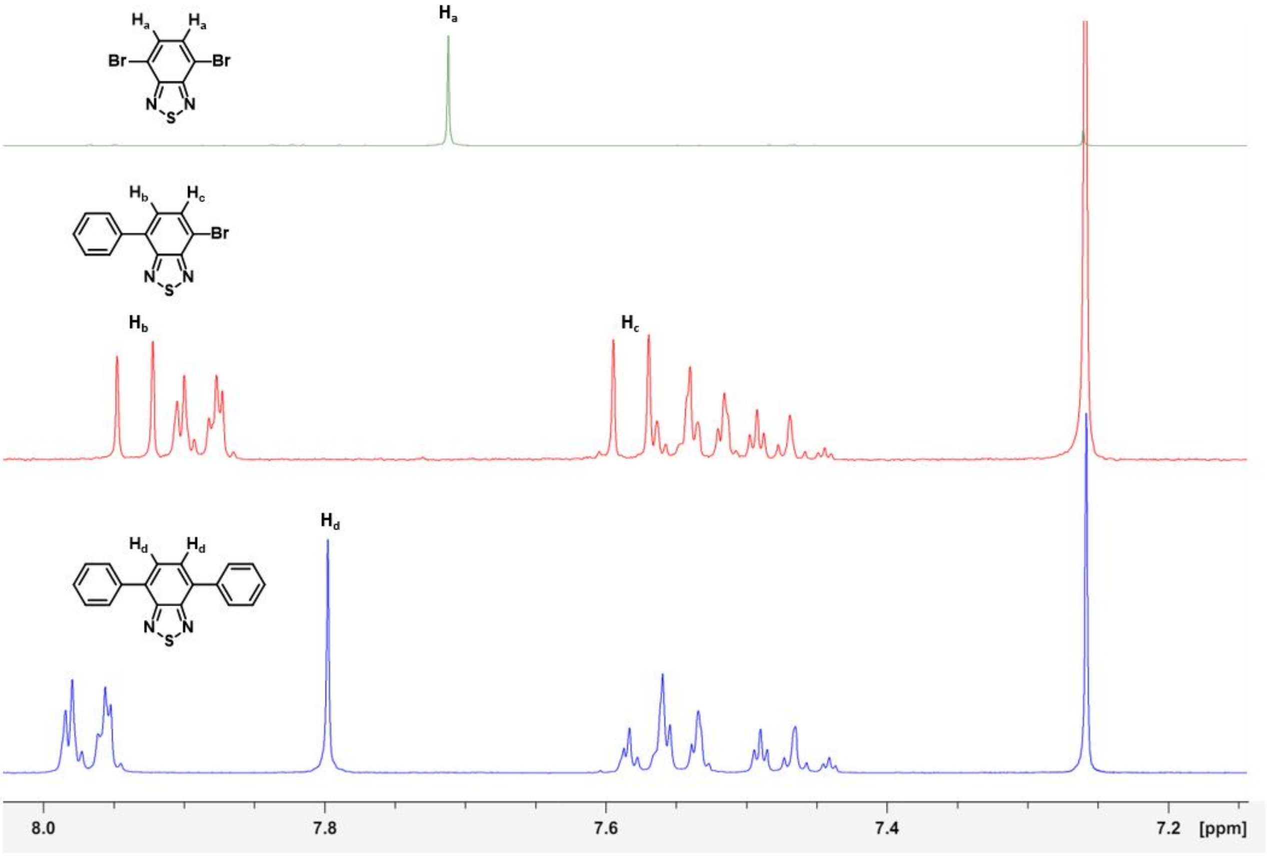

Figure S3 Stacked ^{1}H NMR of pure **Br$_2$BTZ** (top), photocatalyst **1** (bottom), and the intermediate product after a single cross coupling (center, **1-int**) in $CDCl_3$ showing the peaks that can be used to track the course of the Suzuki-Miyaura coupling reaction.

Synthetic Details for 1 – 5[3]

4,7-Diphenylbenzo[c][1,2,5]thiadiazole (1)

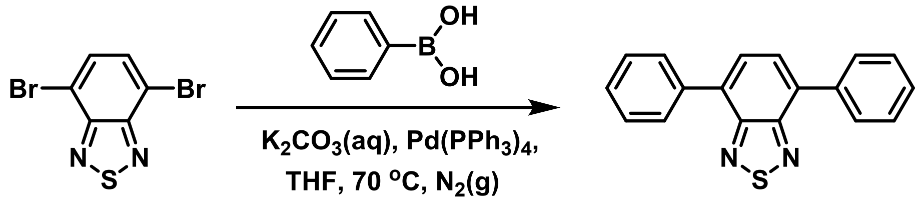

Synthesised using the general procedure for Suzuki-Miyaura coupling using benzene boronic acid (305 mg, 2.5 mmol), **Br_2BTZ** (294 mg, 1.0 mmol), potassium carbonate (276 mg, 2.0 mmol) and $Pd(PPh_3)_4$ (2 mol%). The crude product was recrystallised from hot ethanol to give bright yellow needles (173 mg, 60%). **^{1}H NMR** ($CDCl_3$, 300 MHz, 25.0 °C) δ_H 7.97 (m, 4 *H*), 7.80 (s, 2 *H*), 7.59 (m, 4 *H*), 7.47 (m, 2 *H*). **^{13}C NMR** ($CDCl_3$, 75.5 MHz, 25.0 °C) δ_C 154.1 (C), 137.5 (C), 133.4 (C), 129.3 (CH), 128.7 (CH), 128.4 (CH), 128.2 (CH). **UV-Vis** ($CHCl_3$) λ_{max} (nm) 380. **IR** $\bar{\nu}$ (cm^{-1}) 3060 (w, C-H str.).

4,7-Di(thiophen-2-yl)benzo[c][1,2,5]thiadiazole (2)

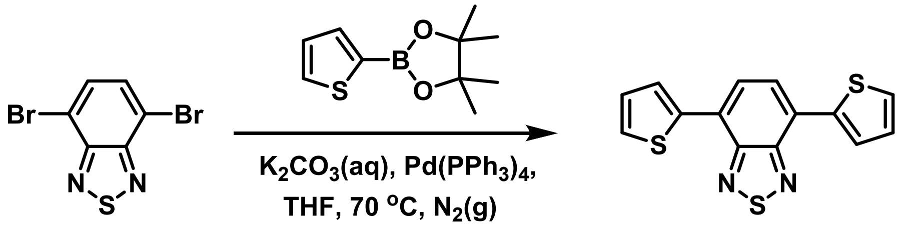

Synthesised using the general procedure for Suzuki-Miyaura coupling using 2-thiophene boronic acid pinacol ester (320 mg, 2.5 mmol), **Br_2BTZ** (294 mg, 1.0 mmol), potassium carbonate (276 mg, 2.0 mmol) and $Pd(PPh_3)_4$ (5 mol%). The crude product was recrystallised from hot ethanol to give red needles (200 mg, 67%). **^{1}H NMR** ($CDCl_3$, 300 MHz, 25.0 °C) δ_H 8.13 (dd, J = 3.7, 1.1 Hz, 2 *H*), 7.89 (s, 2 *H*), 7.45 (dd, J = 5.1, 1.1 Hz, 2 *H*), 7.22 (dd, J = 5.1 Hz, 3.7 Hz, 2 *H*). **^{13}C NMR** ($CDCl_3$, 75.5 MHz, 25.0 °C) δ_C 152.7 (C), 139.4 (C), 128.0 (CH), 127.5 (CH), 126.8 (CH), 126.1 (C), 125.8 (CH). **UV-Vis** ($CHCl_3$) λ_{max} (nm) 446. **IR** $\bar{\nu}$ (cm^{-1}) 2990 (w, C-H str.).

4,7-Di([1,1'-biphenyl]-2-yl)benzo[c][1,2,5]thiadiazole (3)

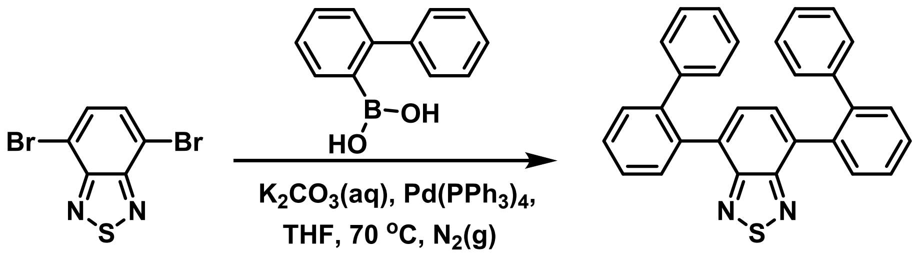

Synthesised using the general procedure for Suzuki-Miyaura coupling using 2-biphenyl boronic acid (495 mg, 2.5 mmol), **Br_2BTZ** (294 mg, 1.0 mmol), potassium carbonate (276 mg, 2.0 mmol) and $Pd(PPh_3)_4$ (5 mol%). The crude product was washed with ethanol to yield a yellow powder (280 mg, 64%). **^{1}H NMR** ($CDCl_3$, 300 MHz, 25.0 °C) δ_H 7.60 (m, 2 *H*), 7.52 (m, 4 *H*), 7.48 (m, 2 *H*), 7.14 (s, 2 *H*), 7.09 (m, 10 *H*). **UV-Vis** ($CHCl_3$) λ_{max} (nm) 373. **IR** $\bar{\nu}$ (cm^{-1}) 3060 (w, C-H str.). A ^{13}C NMR spectra of suitable quality could not be obtained due to the low solubility of **3** in common deuterated solvents.

4,7-Di(naphthalen-1-yl)benzo[*c*][1,2,5]thiadiazole (4)

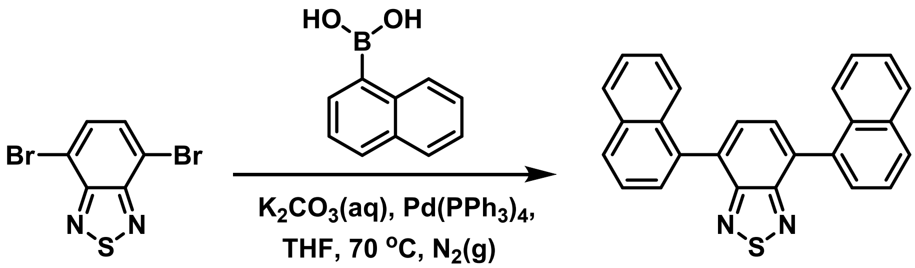

Synthesised using the general procedure for Suzuki-Miyaura coupling using napthalen-1-ylboronic acid (375 mg, 2.5 mmol), **Br_2BTZ** (294 mg, 1.0 mmol), potassium carbonate (276 mg, 2.0 mmol) and $Pd(PPh_3)_4$ (5 mol%). The crude product was washed with hot ethanol to yield a bright yellow powder (340 mg, 87%). **^{1}H NMR** ($CDCl_3$, 300 MHz, 25.0 °C) δ_H 8.00 (m, 2 *H*), 8.00 (m, 2 *H*), 7.84 (s, 2 *H*), 7.73 (m, 2 *H*), 7.73 (m, 2 *H*), 7.66 (m, 2 *H*), 7.55 (m, 2 *H*), 7.44 (m, 2 *H*). **^{13}C NMR** ($CDCl_3$, 75.5 MHz, 25.0 °C) δ_C 154.8 (C), 135.5 (C), 133.9 (C), 133.4 (C), 131.9 (C), 130.3 (CH), 129.0 (CH), 128.6 (CH), 128.1 (CH), 126.3 (CH), 126.1 (CH), 125.9 (CH), 125.4 (CH). **UV-Vis** ($CHCl_3$) λ_{max} (nm) 376. **IR** $\bar{\nu}$ (cm^{-1}) 3050 (w, C-H str.).

4,7-Di(naphthalen-2-yl)benzo[*c*][1,2,5]thiadiazole (5)

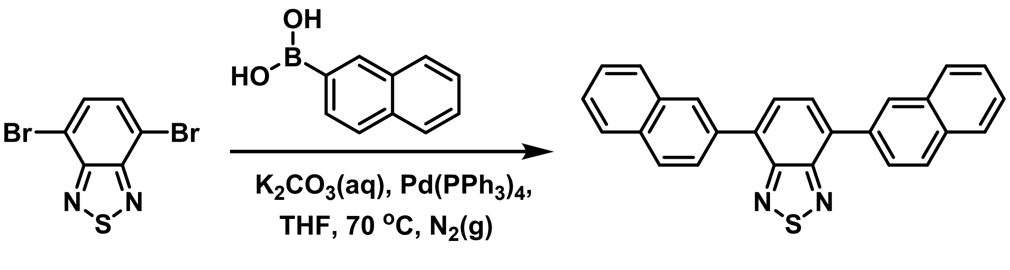

Synthesised using the general procedure for Suzuki-Miyaura coupling using napthalen-2-ylboronic acid (430 mg, 2.5 mmol), **Br_2BTZ** (294 mg, 1.0 mmol), potassium carbonate (276 mg, 2.0 mmol) and $Pd(PPh_3)_4$ (5 mol%). The crude product was washed with hot ethanol to yield a yellow powder (336 mg, 86%). **^{1}H NMR** ($CDCl_3$, 300 MHz, 25.0 °C) δ_H 8.51 (d, J = 1.3 Hz, 2 *H*), 8.12 (dd, J = 8.8, 1.8 Hz, 2 *H*), 8.03 (d, J = 8.8 Hz, 2 *H*), 7.99 (m, 2 *H*), 7.97 (s, 2 *H*), 7.93 (m, 2 *H*), 7.56 (m, 2 *H*), 7.56 (m, 2 *H*). **^{13}C NMR** ($CDCl_3$, 75.5 MHz, 25.0 °C) δ_C 135.5 (C), 133.4 (C), 133.2 (C), 128.7 (CH), 128.6 (CH), 128.5 (CH), 128.2 (CH), 127.7 (CH), 127.0 (CH), 126.6 (CH), 126.4 (CH). **UV-Vis** ($CHCl_3$) λ_{max} (nm) 396. **IR** $\bar{\nu}$ (cm^{-1}) 3050 (w, C-H str.).

1.3 Example Photoredox Procedure

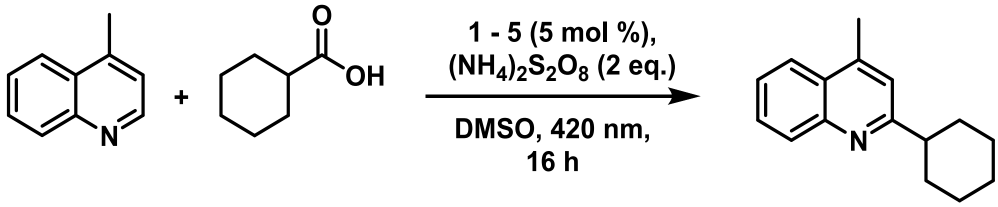

Lepidine (0.3 mmol), photocatalyst (5 mol%), ammonium peroxydisulfate (0.60 mmol), cyclohexane carboxylic acid (3.0 mmol) and DMSO (3 mL) were added to a dried vial. The mixture was degassed by bubbling with nitrogen gas for 10 minutes then the mixture irradiated using a 420 nm LED for 16 hours. Following this time, the mixture was diluted with DCM (10 mL) then washed with saturated sodium carbonate solution (15 mL). The aqueous phase was extracted with DCM (3 x 10 mL) and the combined organic phases dried over $MgSO_4$. The solvent was removed under reduced pressure and the residue dissolved in $CDCl_3$ for analysis by ^{1}H NMR spectroscopy.

Isolation of 2-cyclohexyllepidine can be achieved by silica gel column chromatography (DCM:ethyl acetate 3:1) to yield a yellow oil (50 mg, 73%). **^{1}H NMR** ($CDCl_3$, 300 MHz, 25.0 °C) δ_H 8.05 (dd, J = 8.5, 0.7 Hz, 1 *H*), 7.93 (dd, J = 8.4, 1.0 Hz, 1 *H*), 7.66 (ddd, J = 8.3, 6.8, 1.4 Hz, 1 *H*), 7.48 (ddd, J = 8.4, 6.8, 1.3 Hz, 1 *H*), 7.16 (d, J = 0.7 Hz, 1 *H*), 2.87 (tt, J = 11.9, 3.4 Hz, 3 *H*), 2.67 (d, J = 0.9 Hz, 3 *H*), 2.02 (m, 2 *H*), 1.89 (m, 2 *H*), 1.79 (m, 2 *H*), 1.63 (m, 2 *H*), 1.47 (m, 2 *H*), 1.31 (m, 1 *H*). **^{13}C NMR** ($CDCl_3$, 75.5 MHz, 25.0 °C) δ_C 166.5 (C), 147.7 (C), 144.2 (C), 129.5 (CH), 128.9 (CH), 127.1 (C), 125.4 (CH), 123.6 (CH), 120.3 (CH), 47.6 (CH_3), 32.9 (CH_2), 26.6 (CH_2), 26.2 (CH_2), 18.8 (CH_3).

NOTES TO INSTRUCTORS

- The DMSO used for this reaction does not need to be anhydrous.
- The conversion of this reaction can be determined by ^{1}H NMR of the crude residue dissolved in $CDCl_3$ following work up. Characteristic peaks can be identified for both lepidine and cyclohexyllepidine that can be integrated to determine a conversion (Figure S4). An example NMR showing partial conversion of lepidine into 2-cyclohexyllepidine is shown below with the important peaks highlighted (Figure S5)
- Variables that can be varied in the optimisation of the photocatalytic Minisci coupling include changing the solvent, oxidant, equivalents of cyclohexane carboxylic acid, reaction time, light distance, concentration of the reaction, photocatalyst loading *etc*.
- Homebuilt LEDs were made by mounting 6 x 3 W 410 – 420 nm LEDs (Future Eden Ltd.) onto a 100 x 100 x 40 mm aluminium heatsink (RS Components) using thermal paste (Farnell Ltd., Electrolube TCOR75S). LEDs were connected by soldering to a 600 mA 18-34 V LED driver. For safety, the LED driver was housed inside a watertight plastic enclosure (Figure S6A).

- Alternative light sources can be used including cheap household lightbulbs, however, these provide lower light intensity for the same power and a wider distribution of wavelengths.

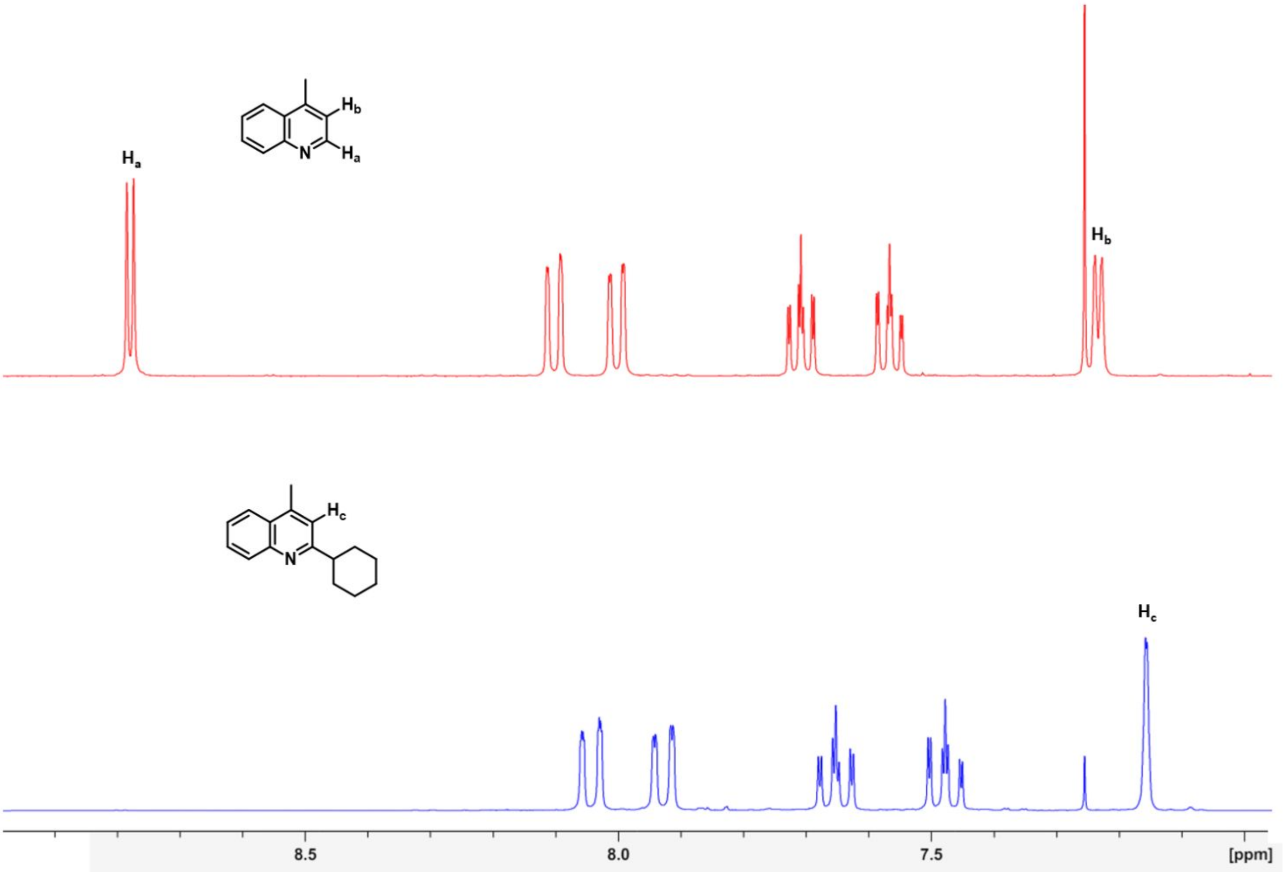

Figure S4 Stacked ^{1}H NMR of pure lepidine (top) and 2-cyclohexyllepidine (bottom) in $CDCl_3$ showing the peaks that can be used to track the course of the Minisci coupling photoredox reaction.

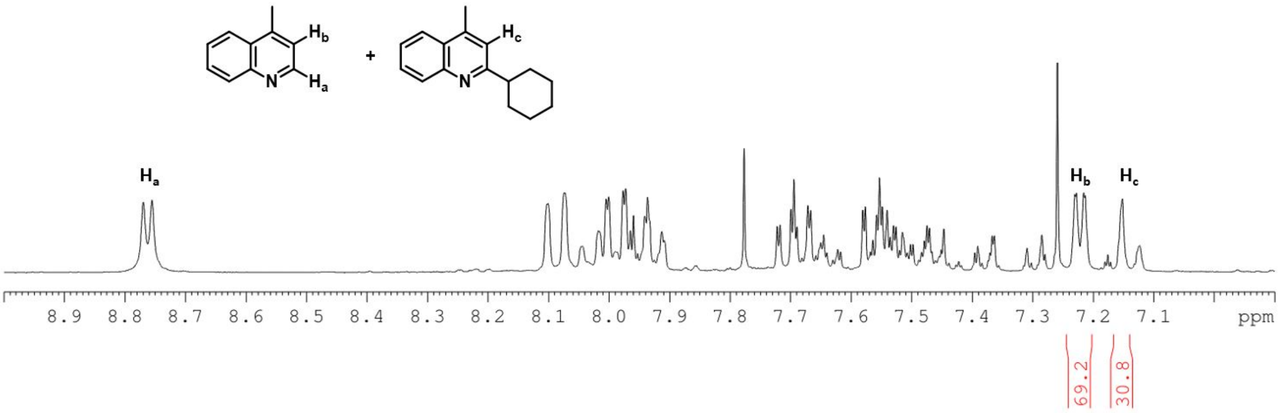

Figure S5 Example of a Minisci photoredox reaction after work up showing partial conversion of lepidine into 2-cyclohexyllepidine. The estimated composition of the worked up reaction mixture is 69% lepidine: 31% 2-cyclohexyllepidine. Reaction conditions – 5 mol% **1** after 8 h.

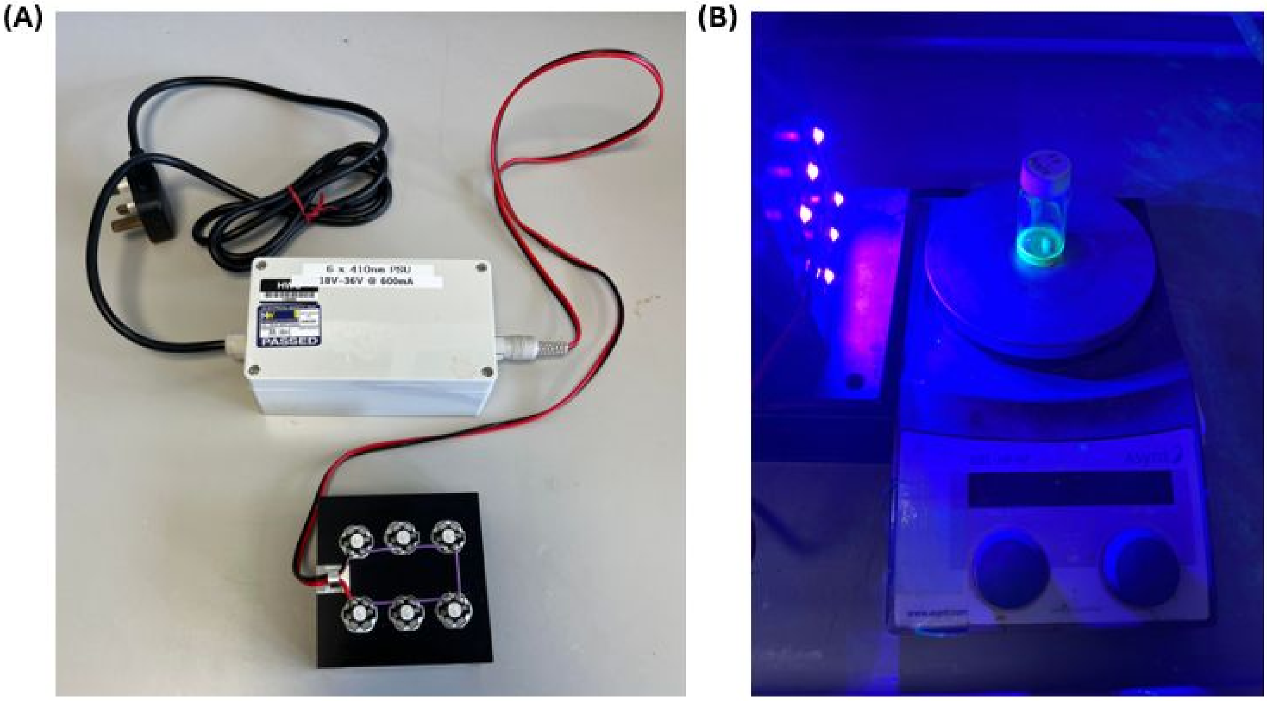

Figure S6 Pictures of experimental apparatus for Minisci coupling photoredox reactions. (A) Picture of the home built LED module used (410-420 nm). (B) Experimental set up consisting of the vial containing the reaction mixture with a magnetic stirrer, magnetic stirring plate and LED module.

1.4 Computational Methods

Energy barriers to rotation about the electron donor-acceptor bond were modelled using Chem3D Pro software using the MM2 force field. The structures were first energy minimised using a MM2 before performing the dihedral driver measurement: the absolute values to the rotational energy barriers were then converted into a relative energy value. Modelling of the HOMO and LUMO for **1** – **5** was achieved using the Hyperchem 8 software package using the ZINDO/s method.

NOTES TO INSTRUCTORS

- The students can investigate the effect that different force fields and basis sets have on the computational calculations. We have obtained reasonable results using MM2 (torsional angles) and ZINDO/s (HOMO/LUMO determination).

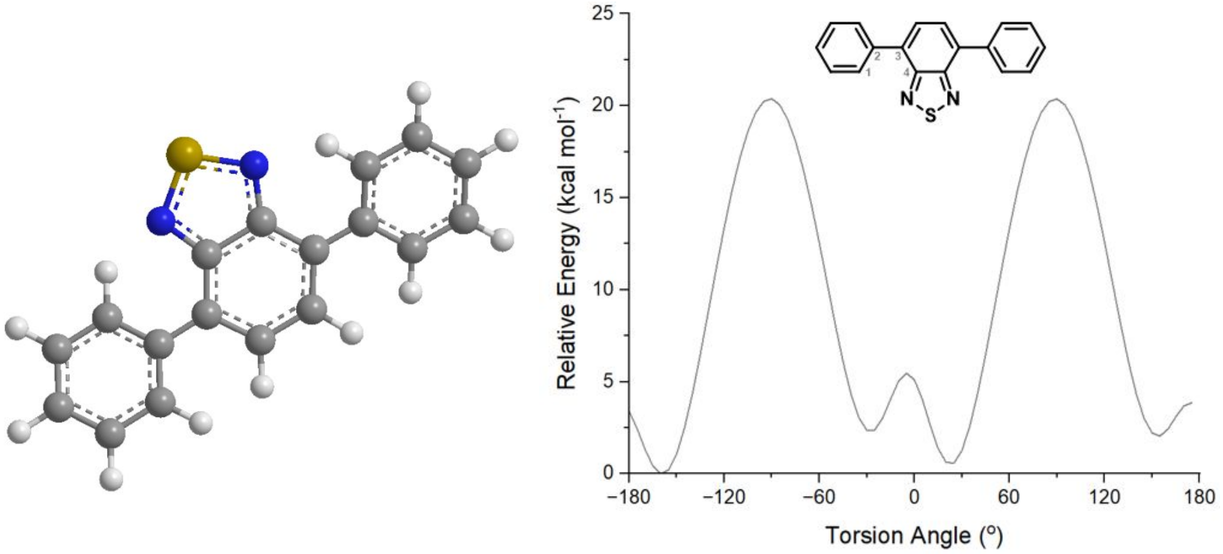

Figure S7 (Left) MM2 minimised 3D model of **1**. (Right) Torsion angle plot for rotation about the highlighted torsion angle.

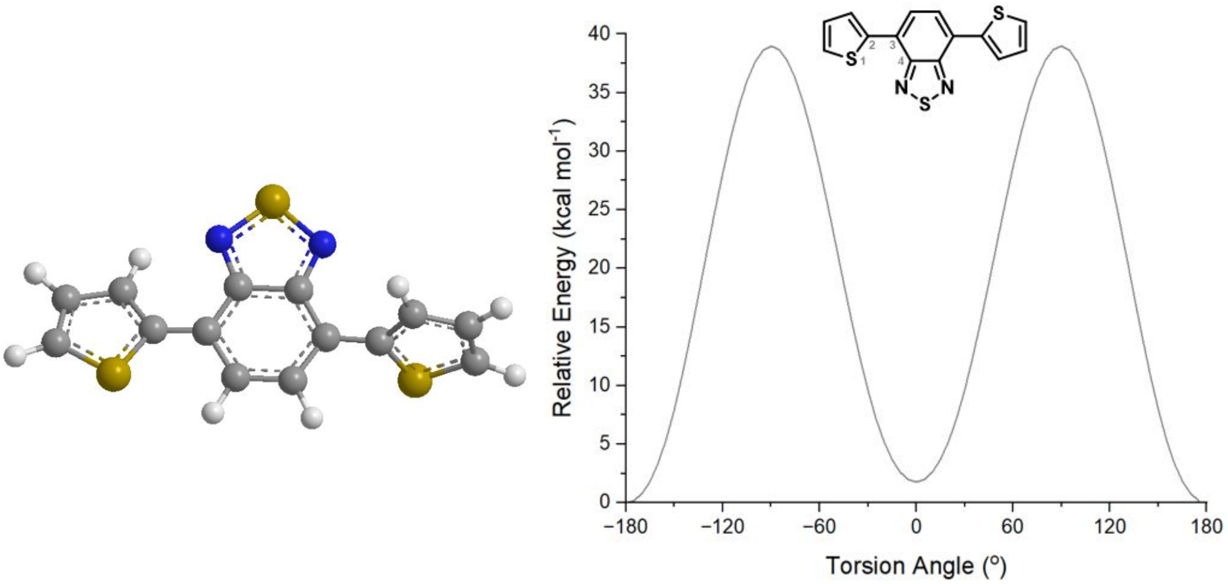

Figure S8 (Left) MM2 minimised 3D model of **2**. (Right) Torsion angle plot for rotation about the highlighted torsion angle.

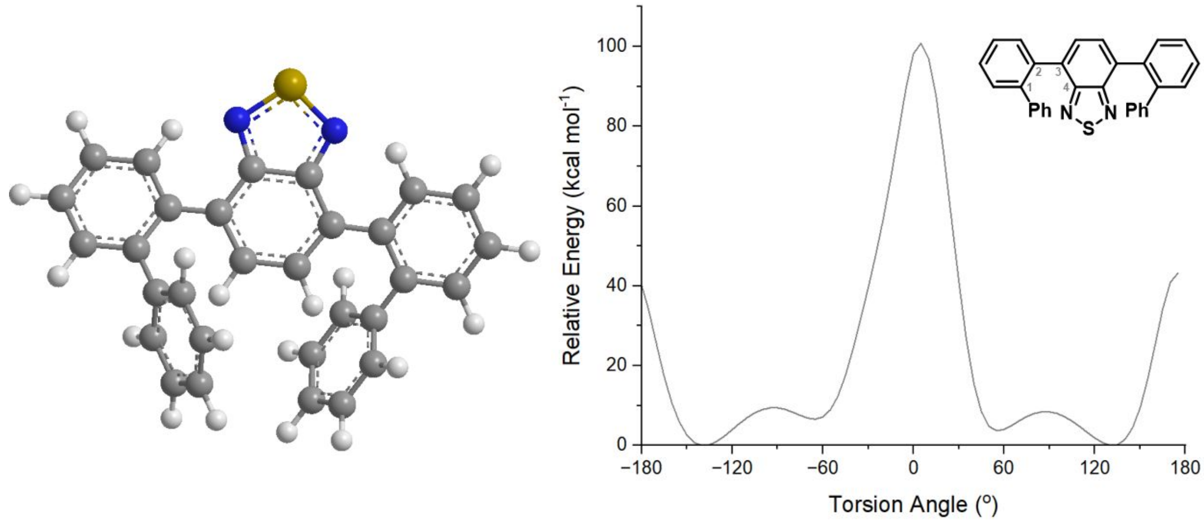

Figure S9 (Left) MM2 minimised 3D model of **3**. (Right) Torsion angle plot for rotation about the highlighted torsion angle.

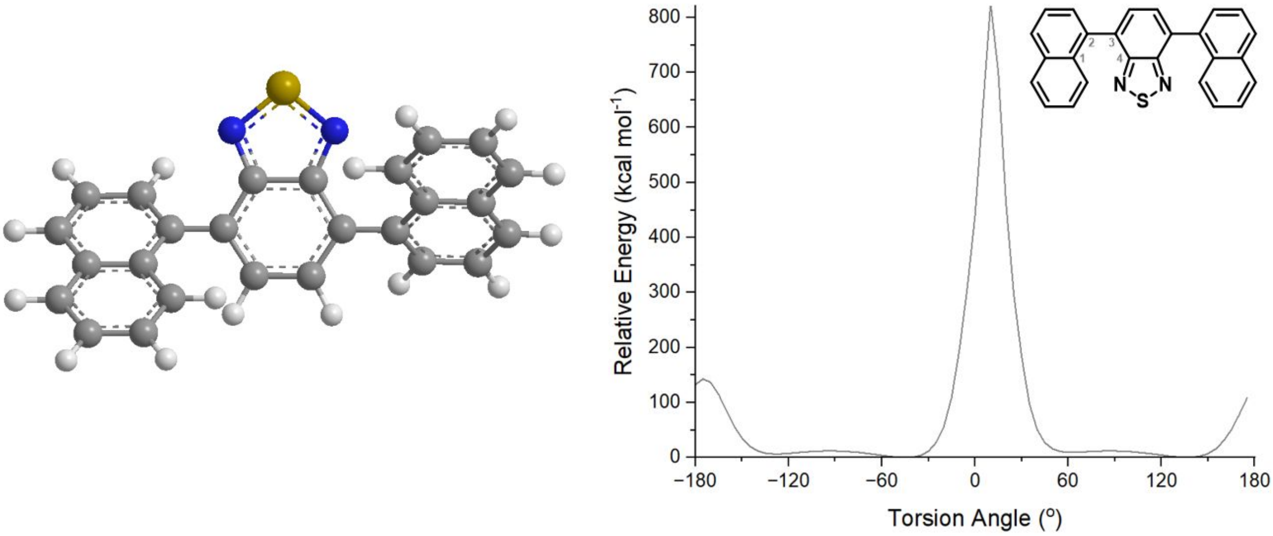

Figure S10 (Left) MM2 minimised 3D model of **4**. (Right) Torsion angle plot for rotation about the highlighted torsion angle.

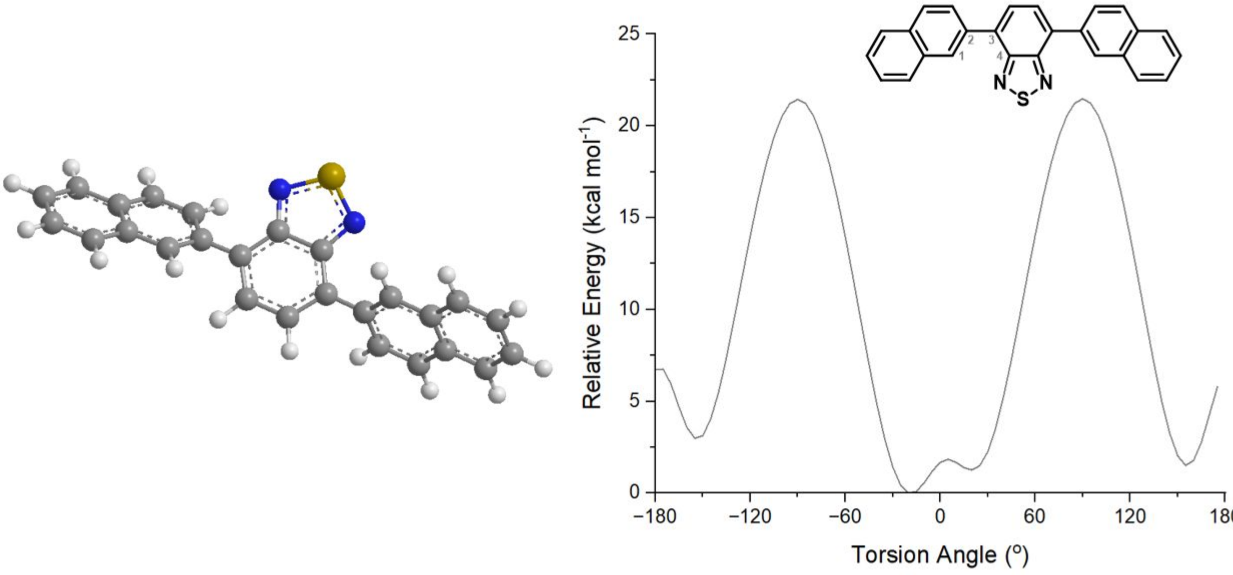

Figure S11 (Left) MM2 minimised 3D model of **5**. (Right) Torsion angle plot for rotation about the highlighted torsion angle.

Table S1 HOMO and LUMOs calculated for **1** – **5** using the ZINDO/s method.

Photocatalyst	HOMO	LUMO
**1**	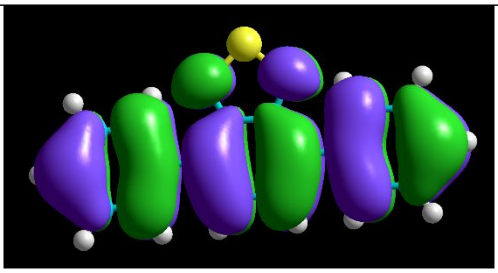	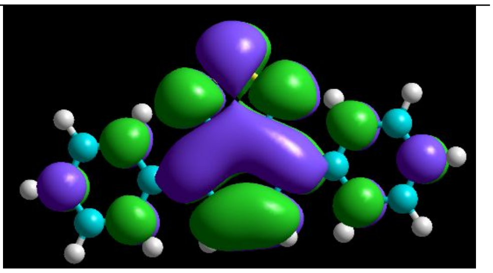
**2**	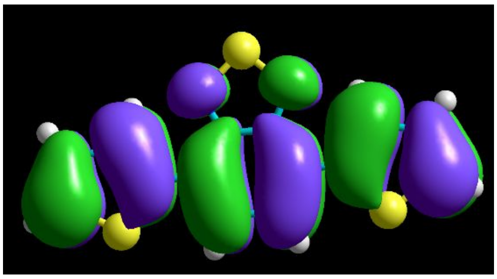	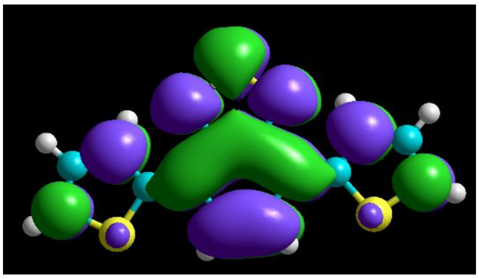
**3**	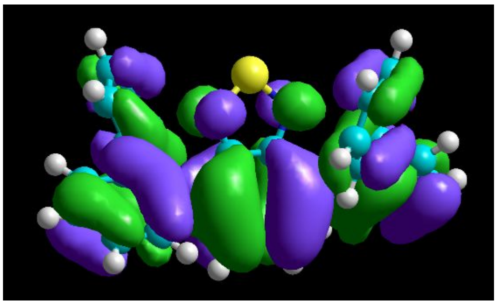	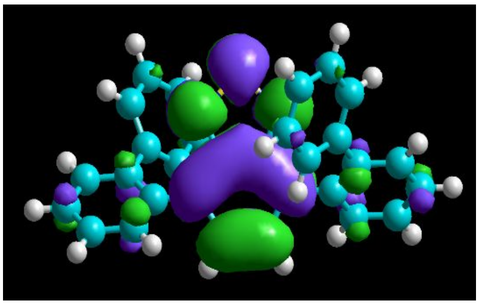
**4**	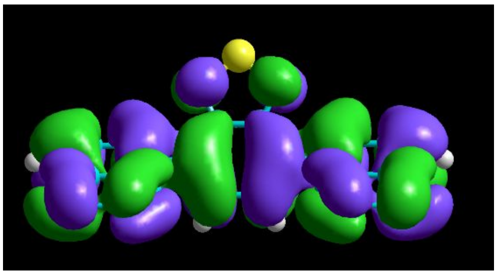	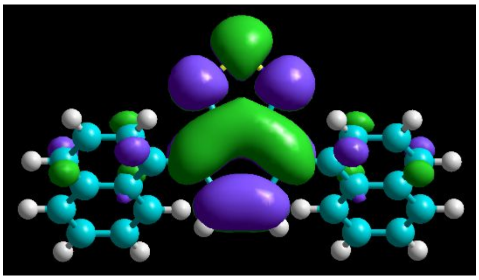
**5**	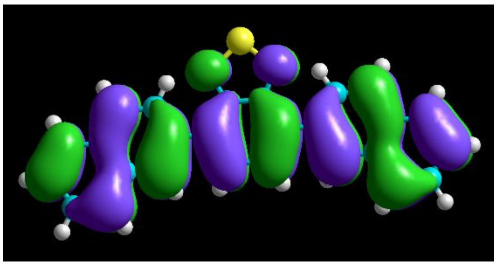	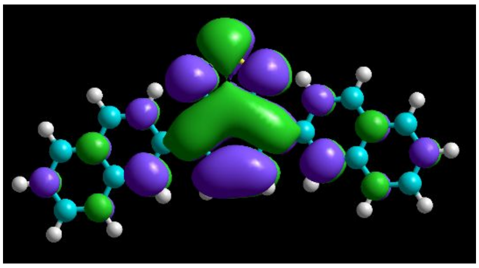

2. Cost Analysis

In this section, an analysis of the cost involved for each section of this miniproject is presented. The calculations of cost performed in this section do not include the cost of electricity (for heating the reaction, vacuum pumps, powering the LED modules *etc.*), common laboratory solvents (methanol, diethyl ether, THF or DCM), cost of common laboratory equipment (hotplates, glassware *etc.*), nitrogen gas, or cost of any labour. The suppliers listed are those from which the chemicals were purchased to run this project at Heriot-Watt University. All purchases were made using the British Pound Sterling (GBP, £): for the cost calculations these values have been converted into US Dollars (USD, $) using a conversion rate of £0.74 = $1 (accurate as of 26th September 2024).

4,7-Dibromobenzo[*c*][1,2,5]thiadiazole (Br_2BTZ)

Table S2 Cost analysis for **Br_2BTZ**.

Chemical	Chemical Supplier	Amount Purchased	Cost/ £	Cost/ $	Amount Used	Cost of Amount Used/$
Benzo[c][1,2,5]thiadiazole	Fluorochem	100 g	45.00	60.81	2.50 g	1.53
N-Bromosuccinimide	Sigma-Aldrich	1 kg	38.50	52.03	6.88 g	0.35
98% Sulfuric Acid	Fischer Scientific	1 L	43.70	59.05	25 mL	1.47

Total cost per synthesis of **Br_2BTZ** = $3.35

Mass of **Br_2BTZ** obtained = 4.52 g

Cost per gram of **Br_2BTZ** = $0.74 g^{-1} = £0.55 g^{-1}

4,7-Diphenylbenzo[*c*][1,2,5]thiadiazole (1)

Table S3 Cost analysis for **1**. [a]Cost of THF, DCM, water and $MgSO_4$ not included as these were readily available in the teaching laboratory. [b]Cost of **Br_2BTZ** based on the calculation shown above.

Chemical[a]	Chemical Supplier	Amount Purchased	Cost/ £	Cost/ $	Amount Used	Cost of Amount Used/$
**Br_2BTZ**[b]	-	4.52 g	2.48	3.35	0.294 g	0.22
Benzene boronic acid	Fluorochem	25 g	16.00	21.62	0.305 g	0.27
$Pd(PPh_3)_4$[b]	Fluorochem	1 g	17.00	22.97	0.023 g	0.53
Potassium carbonate	Fluorochem	500 g	14.00	18.92	0.276 g	0.01

Total cost per synthesis of **1** = $1.03

Mass of **1** obtained = 0.173 g

Cost per gram of **1** = $5.89 g^{-1} = £4.36 g^{-1}

Table S4 Summary of the cost of synthesising photocatalysts **1** – **5** using the method shown in Table S3.

Photocatalyst	Boronic Acid Supplier	Amount Purchased	Cost/ £	Cost/ $	Cost per synthesis/ $	Yield of Photocatalyst/ mg	Cost of photocatalyst per gram/ $ g^{-1}
**1**	Fluorochem	25 g	16.00	21.62	1.03	173	5.89
**2**	Fluorochem	25 g	25.00	33.78	2.19	200	10.95
**3**	Fluorochem	25 g	30.00	40.54	2.27	280	8.11
**4**	Fluorochem	25 g	16.00	21.62	2.04	340	6.00
**5**	Fluorochem	25 g	22.00	29.73	2.14	336	6.36

Cost of Performing a Minisci Coupling

Table S5 Cost analysis for a typical Minisci coupling reaction excluding the photocatalyst. [a]Calculated using the density of DMSO at room temperature.

Chemical	Chemical Supplier	Amount Purchased	Cost/ £	Cost/ $	Amount Used	Cost of Amount Used/$
Cyclohexane carboxylic acid	Fluorochem	100 g	14.00	18.92	385 mg	0.07
Ammonium peroxydisulfate	Fischer Scientific	500 g	31.10	42.03	137 mg	0.01
Lepidine	Fluorochem	25 g	57.00	77.03	43 mg	0.14
DMSO	Fluorochem	500 g	18.81	25.42	3 mL (3.3 g)[a]	0.16

Typical cost per Minisci coupling = $0.38

Typical number of Minisci photoredox reactions = 14 per group

Total cost of Minisci Coupling testing per group = $5.38

Cost of Assembling a Home Built LED

Table S6 Cost analysis for a building a set of six 410 nm LED modules.

Component	Supplier (Catalogue Number)	Cost (unit size)/£	Cost/$	Amount Used	Cost of Amount Used/$
3 W 410 nm LED with 20mm PCB	Ebay (234048125027)	15.95 (50 LEDs)	21.55	36 LEDs	15.51
Farnell electrolube TCOR75S	Farnell UK (1743753)	39.10 (1 tube - 75 mL)	52.84	1 tube	52.84
Heatsink Universal Square Alu, 1K/W, 100 x 100 x 40 mm.	RS Components Ltd. (189-8274)	13.86 (1 unit)	18.73	6 units	112.38
600 mA 18-34 V Aluminium IP67 LED Driver Power Supply	Chanzon *via* Ebay (275755539367)	12.25 (1 unit)	16.55	6 units	99.32
HAMMOND 1554JGY, Plastic Enclosure, Watertight, DIN Rail, ABS, 61 mm, 89 mm, 160 mm, IP66	Farnell UK (4081020)	13.34 (1 unit)	18.03	6 units	108.16
MULTICOMP PRO PG9DG, Cable Gland, With Locknut, IP65, PG9, 4 mm, 8 mm, Nylon (Polyamide), Grey (Pack of 10)	Farnell UK (1621062)	3.71 (10 pack)	5.01	1 pack	5.01
HIRSCHMANN MAB 3100 S DIN Audio / Video Connector, 3 Contacts, Jack, Panel Mount, Solder, Tin Plated Contacts	Farnell UK (496418)	1.93 (1 unit)	2.61	6 units	15.65
HIRSCHMANN MAS 3100 DIN Audio / Video Connector, 3 Contacts, Plug, Cable Mount, Solder, Tin Plated Contacts	Farnell UK (496820)	2.35 (1 unit)	3.18	6 units	19.05
VOLEX X-143994A Mains Power Cord, With Fuse, Mains Plug, UK to Free End, 2 m, 5 A, 250 VAC, Black	Farnell UK (1124371)	7.05 (1 unit)	9.53	6 units	57.24

Number of LED modules prepared = 6

Overall cost = $485.17

Cost per LED Module = $80.86 = £59.84

3. ^{1}H NMR Spectra of Pure Starting Materials and Products

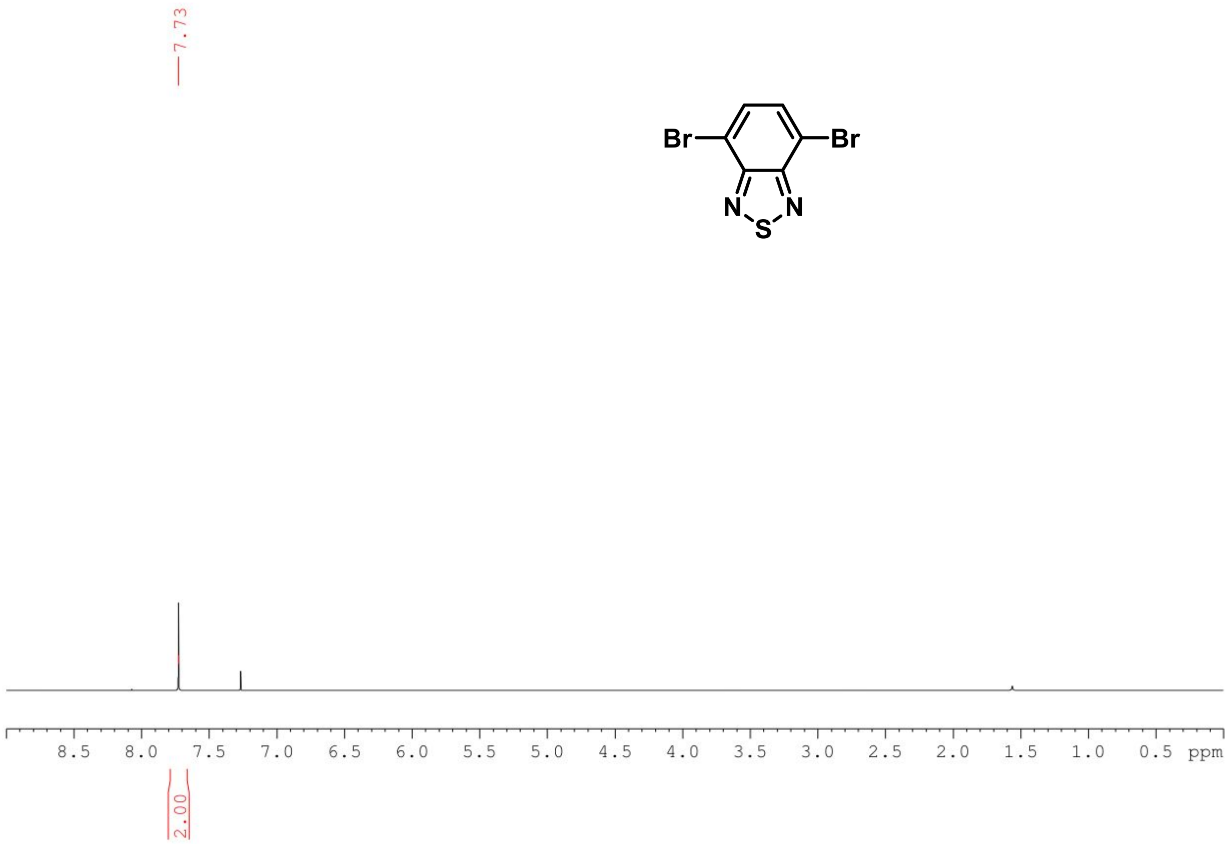

Figure S12 ^{1}H NMR spectra of **Br_2BTZ** in $CDCl_3$.

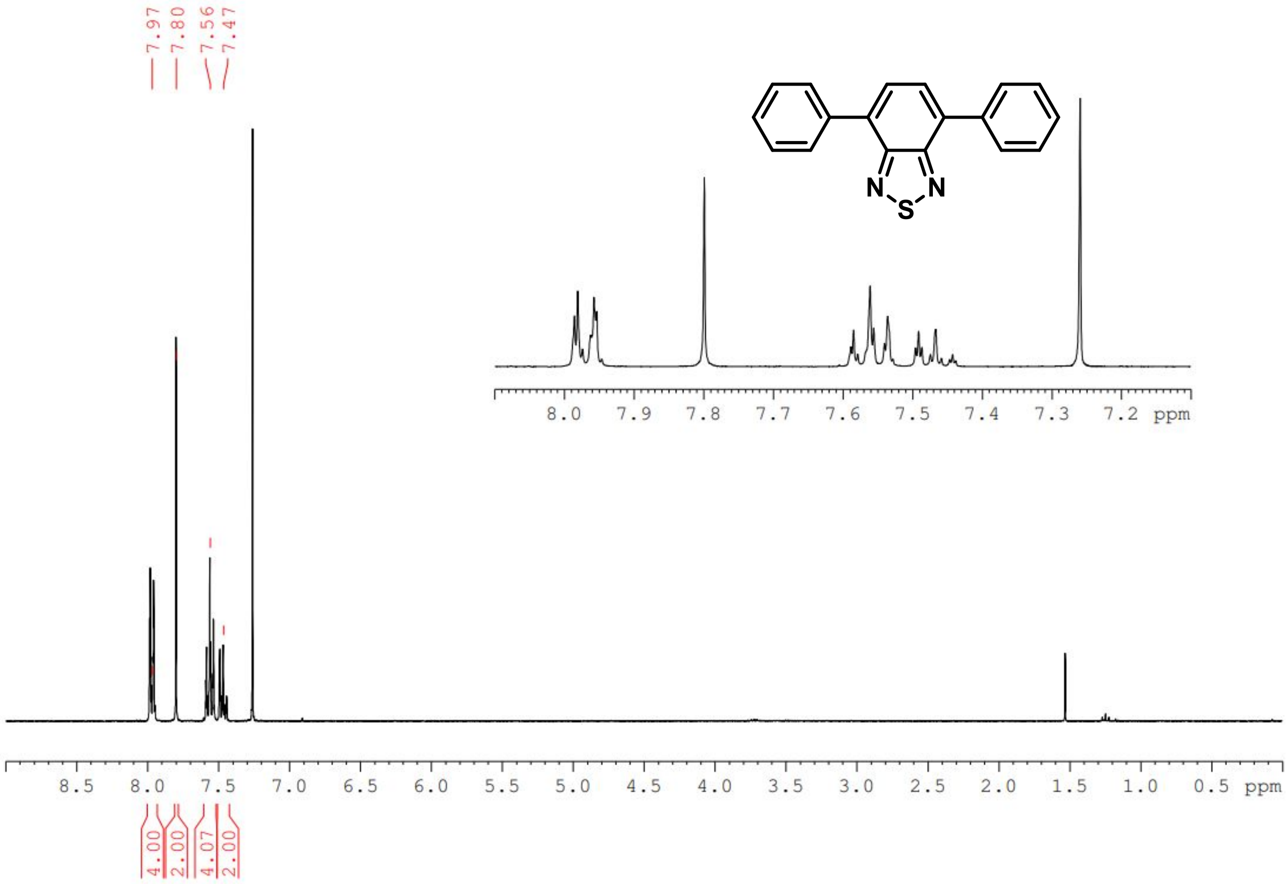

Figure S13 ^{1}H NMR spectra of **1** in $CDCl_3$

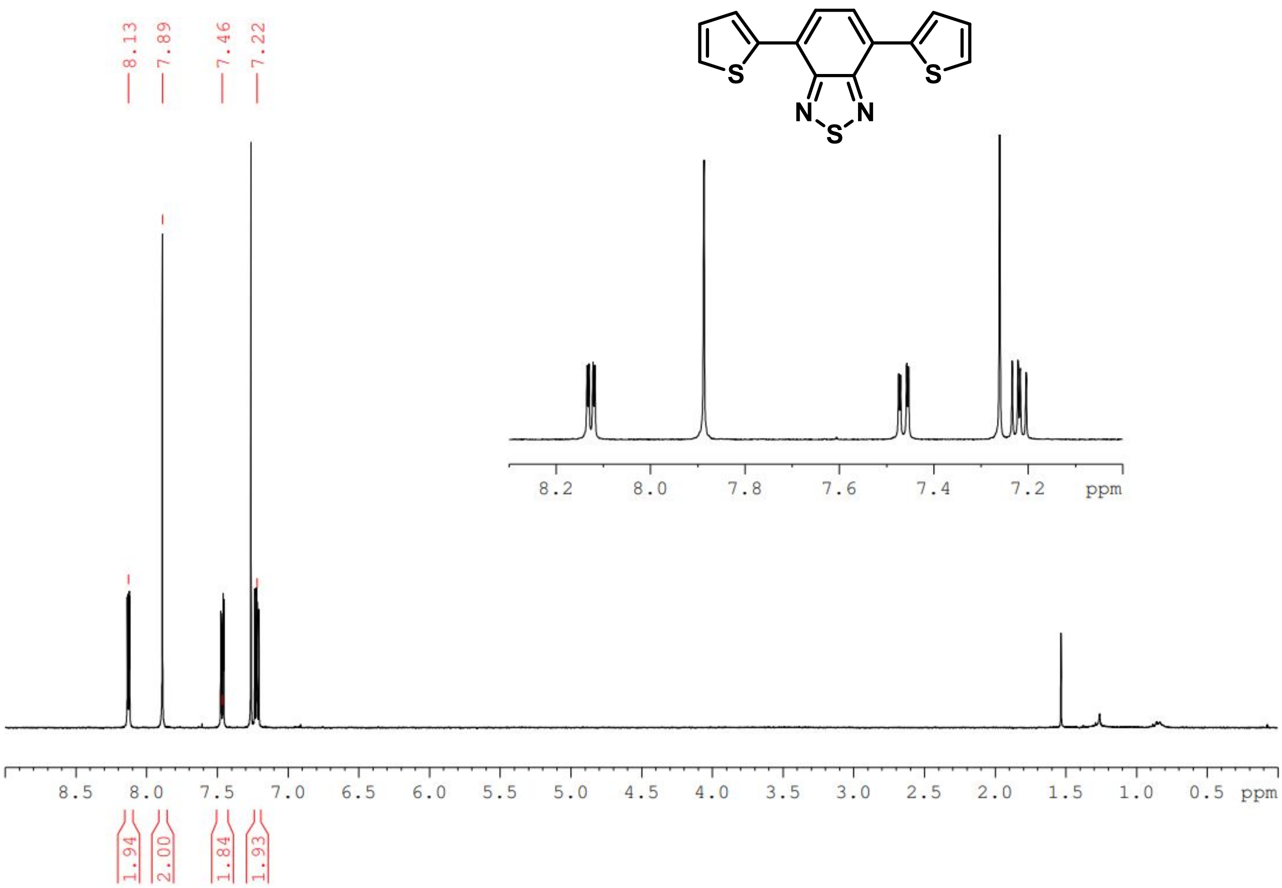

Figure S14 ^{1}H NMR spectra of **2** in $CDCl_3$.

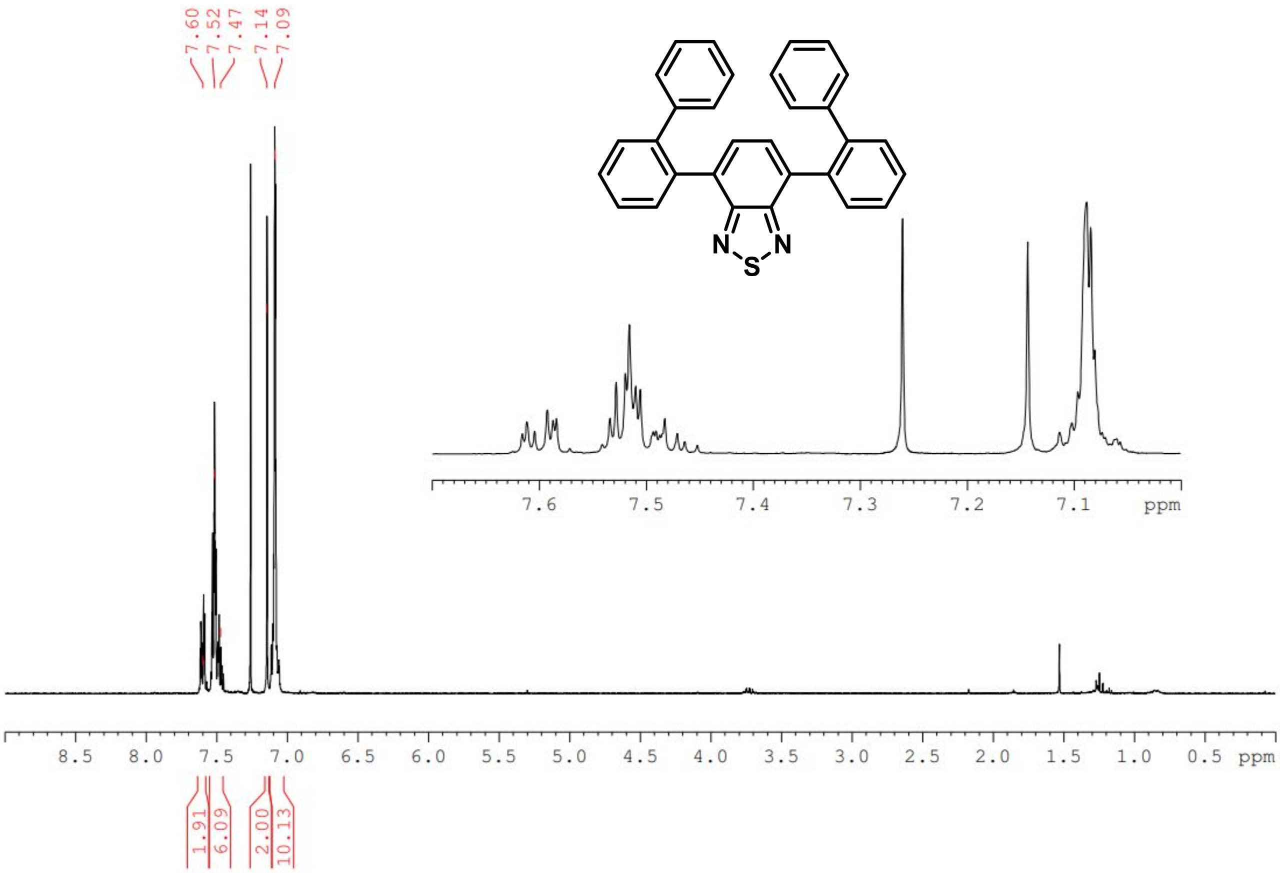

Figure S15 ^{1}H NMR spectra of **3** in $CDCl_3$

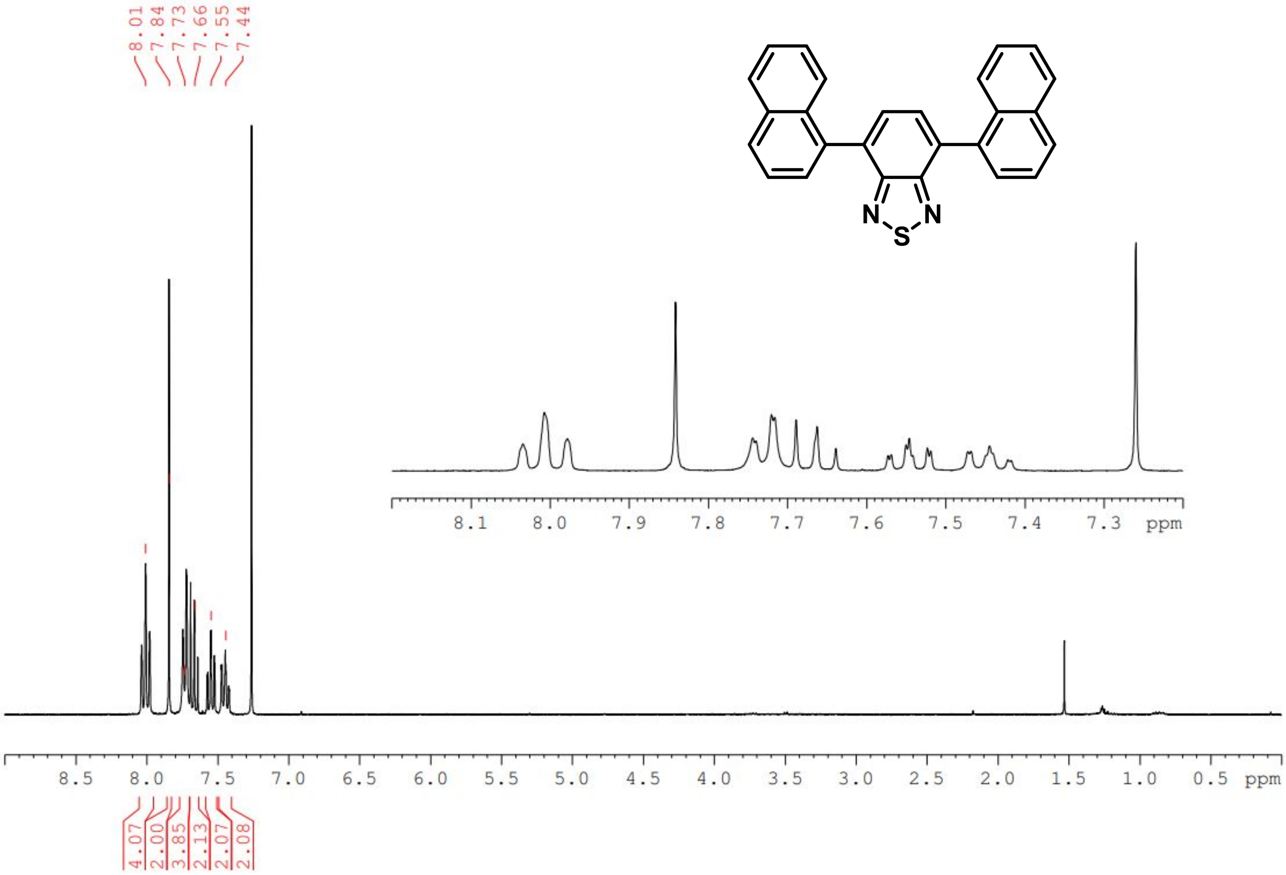

Figure S16 ^{1}H NMR spectra of **4** in $CDCl_3$.

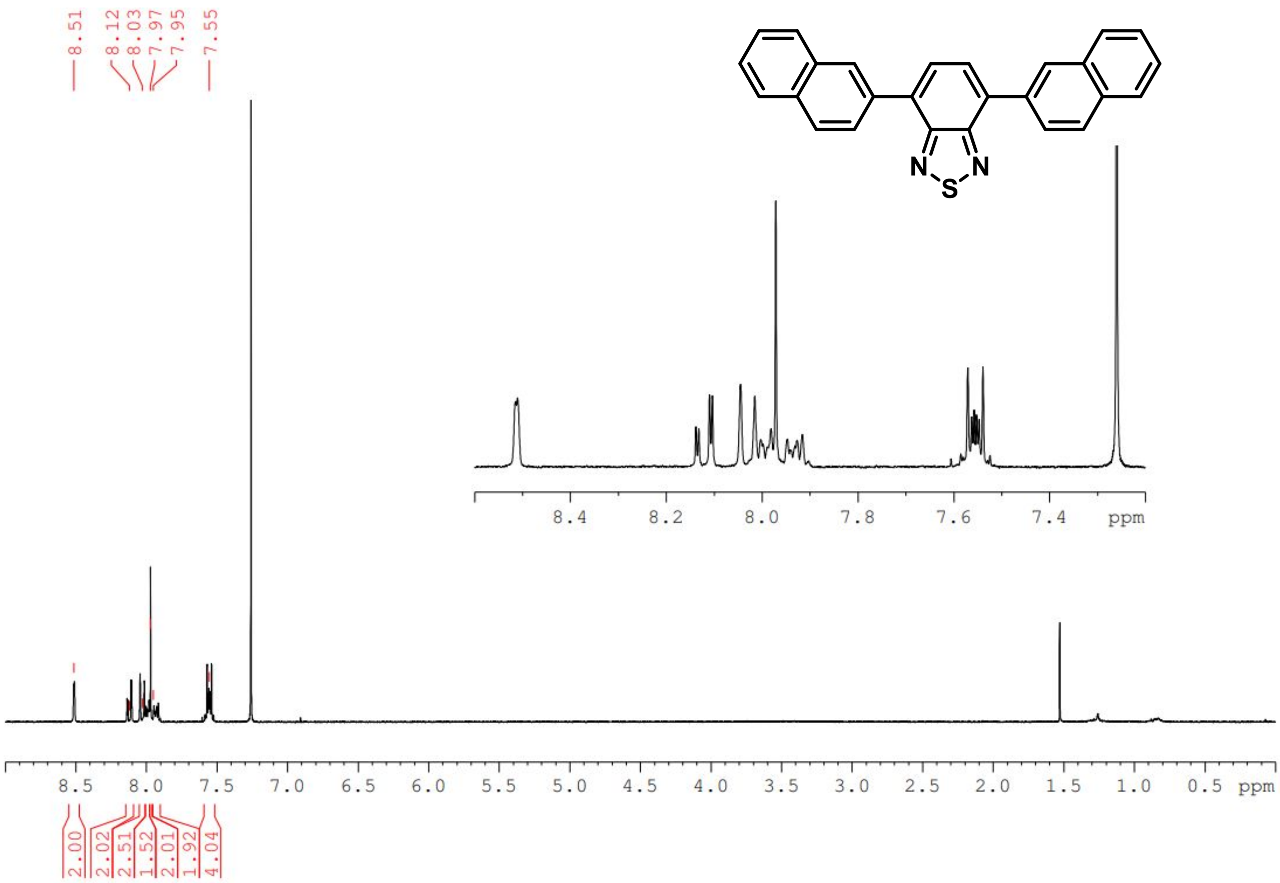

Figure S17 ^{1}H NMR spectra of **5** in $CDCl_3$.

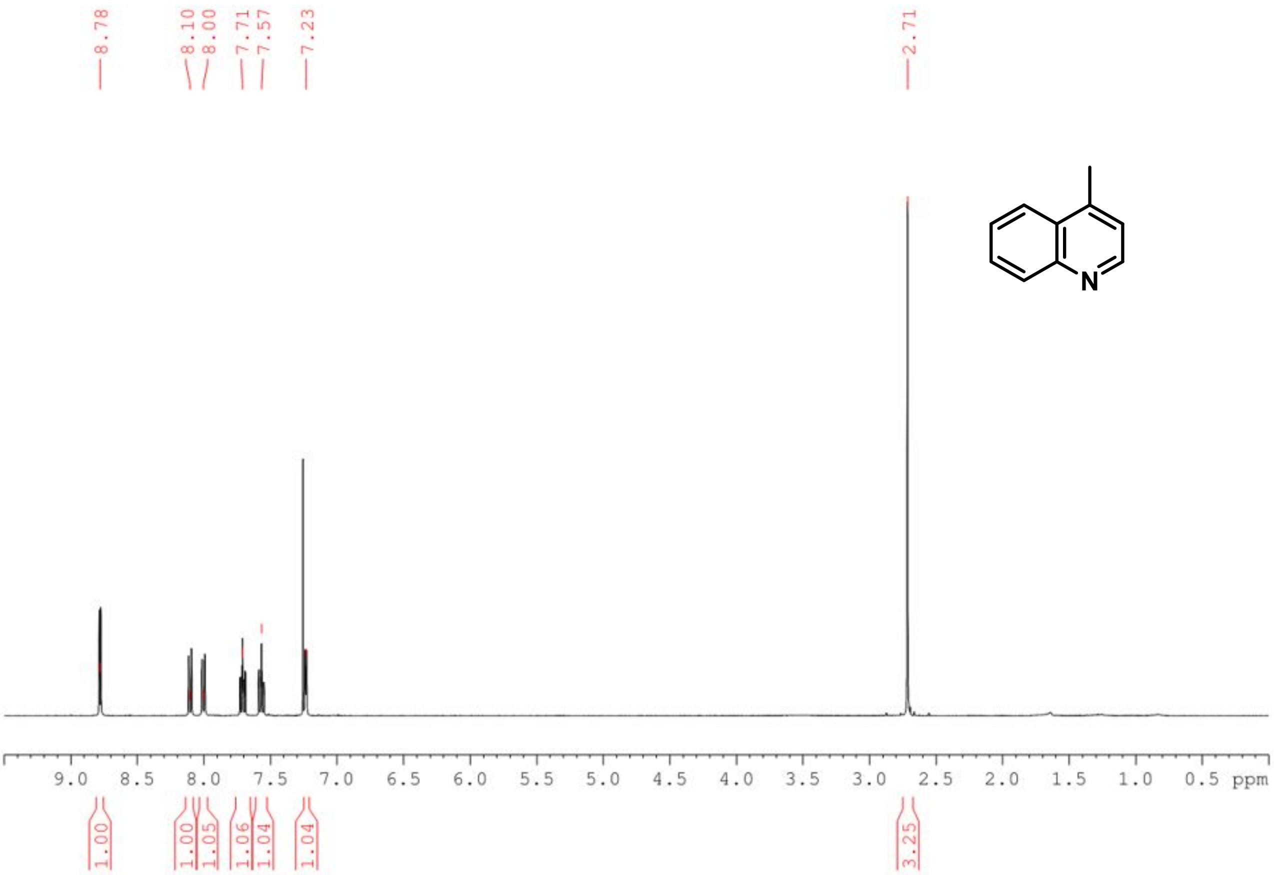

Figure S18 ^{1}H NMR spectra of lepidine in $CDCl_3$.

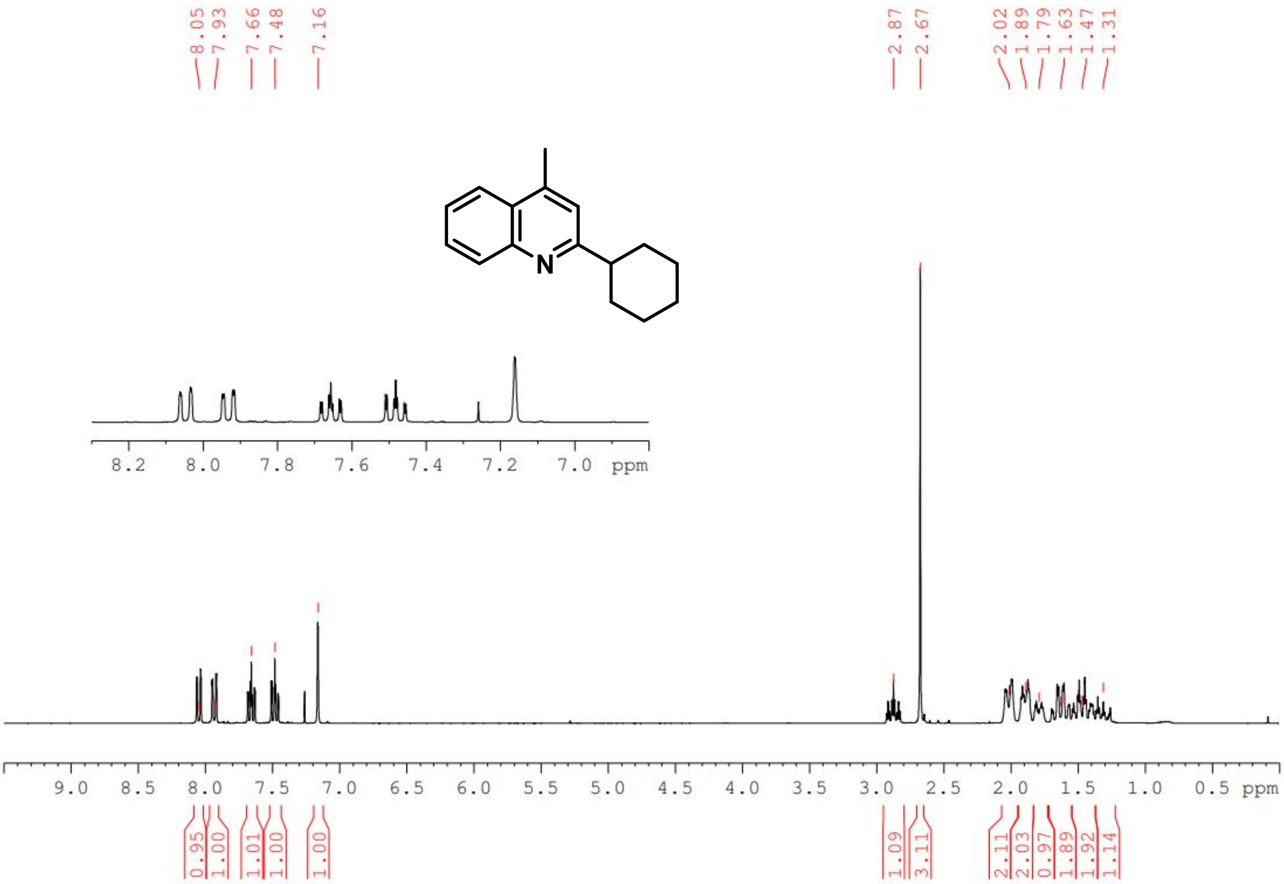

Figure S19 ^{1}H NMR spectra of 2-cyclohexyllepidine in $CDCl_3$.

4. References

1 J. P. Heiskanen, P. Vivo, N. M. Saari, T. I. Hukka, T. Kastinen, K. Kaunisto, H. J. Lemmetyinen and O. E. O. Hormi, *J. Org. Chem.*, 2016, **81**, 1535–1546.

2 B. A. DaSilveira Neto, A. S. A. Lopes, G. Ebeling, R. S. Gonçalves, V. E. U. Costa, F. H. Quina and J. Dupont, *Tetrahedron*, 2005, **61**, 10975–10982.

3 D. Taylor, T. Malcomson, A. Zhakeyev, S.-X. Cheng, G. M. Rosair, J. Marques-Hueso, Z. Xu, M. J. Paterson, S. J. Dalgarno and F. Vilela, *Org. Chem. Front.*, 2022, **9**, 5473–5484.
